# Antiviral activity of the mineralocorticoid receptor NR3C2 against Herpes simplex virus Type 1 (HSV-1) infection

**DOI:** 10.1038/s41598-018-34241-w

**Published:** 2018-10-26

**Authors:** Jürgen G. Haas, Julia Weber, Orland Gonzalez, Ralf Zimmer, Samantha J. Griffiths

**Affiliations:** 1 Division of Infection and Pathway Medicine, University of Edinburgh, Edinburgh, EH16 4SB UK; 2 Institute for Informatics, Ludwig-Maximilians Universität München, 80333 München, Germany

## Abstract

Analysis of a genome-scale RNA interference screen of host factors affecting herpes simplex virus type 1 (HSV-1) revealed that the mineralocorticoid receptor (MR) inhibits HSV-1 replication. As a ligand-activated transcription factor the MR regulates sodium transport and blood pressure in the kidney in response to aldosterone, but roles have recently been elucidated for the MR in other cellular processes. Here, we show that the MR and other members of the mineralocorticoid signalling pathway including HSP90 and FKBP4, possess anti-viral activity against HSV-1 independent of their effect on sodium transport, as shown by sodium channel inhibitors. Expression of the MR is upregulated upon infection in an interferon (IFN) and viral transcriptional activator VP16-dependent fashion. Furthermore, the MR and VP16, together with the cellular co-activator Oct-1, transactivate the hormone response element (HRE) present in the MR promoter and those of its transcriptional targets. As the MR induces IFN expression, our data suggests the MR is involved in a positive feedback loop that controls HSV-1 infection.

## Introduction

Herpes simplex virus type 1 (HSV-1) is a neurotropic α-herpesvirus infecting over 90% of the global population. Whilst HSV-1 is predominantly responsible for vesicular oral or genital skin lesions, it can also cause severe cerebral and eye infections such as meningitis, encephalitis, and keratoconjunctivitis^1,2^. HSV-1 establishes symptomatic lytic infection in epithelial cells as well as an asymptomatic latent infection in trigeminal and sacral ganglia sensory neurons, which can undergo periodic reactivation^3^. The equilibrium between these infection states is dependent upon the interplay between host immunity and viral immune evasion mechanisms, which in turn is determined by a complex network of virus-host interactions^4^. We have previously used genome-scale screening strategies, including yeast two-hybrid (Y2H) protein interaction and RNA interference screens, to gain a comprehensive overview of host factors influencing HSV-1 replication and pathogenesis^5^. This combined screening approach identified several protein families where one member displayed the opposite functional phenotype to most other proteins in this family. One such example was the nuclear receptor superfamily, a group of transcription factors that directly activate gene expression following ligand binding. This group includes receptors for metabolites, such as bile acids, fatty acids, and oxysterols, and steroid hormones, like androgens, progesterone, and corticosteroids, which can be subdivided into glucocorticoid (GC) and mineralocorticoid (MC).

The nuclear receptor NR3C2 is the mineralocorticoid receptor (MR), and whilst it is expressed in a broad range of cell types, including the gastrointestinal tract, immune cells, brain, heart, bone, skin and skeletal muscle, the main target for its ligand mineralocorticoid is polarised epithelial cells^6^. In these cells, the cytoplasmic complex of ligand:MR and chaperone proteins (HSP90 and the immunophilin FKBP4) translocates to the nucleus via the dynein microtubule network, where the MR induces expression of genes involved in sodium transport, such as the serum/glucocorticoid regulated kinase 1 (SGK1)^7^. SGK1 phosphorylates the ubiquitin ligase Nedd4L to reduce its interaction with the epithelial sodium channel (ENaC). This consequently increases cell surface expression of the ENaC and thus sodium reabsorption across the apical membrane, enabling regulation of blood pressure in response to aldosterone^8–10^.

Whilst the closely-related GCs have many cellular, largely anti-inflammatory, functions such as influencing cytokine expression and interferon responses, and modulating helper and cytotoxic T-cell, MK and DC function, the regulation of sodium transport was considered the major function of the MR and downstream MC signalling network^11,12^. Over recent years significant roles for the MR have been identified in non-epithelial tissues, including an effect on memory/affect in the hippocampus^13,14^, on fat biology in adipocytes^15,16^, and in hypertension and cardiac fibrosis in the cardiovascular system^17^. More recently, SGK1, a major target for MR-responsive transcription, has been identified as a key regulator of T-cell differentiation mediated by the metabolic checkpoint kinase complex mTORC2^18^. In this pathway, SGK1 simultaneously promotes T_H_2 differentiation whilst inhibiting T_H_1 cytokines, a phenomenon likely to have a significant negative effect on immune responses during viral infection. Furthermore, both the MR and GR mediate sympathectomy-induced alterations of HSV-1-specific CTL function^12^. It is becoming clear that the MR and its downstream signalling pathway may play a much more significant role in the regulation of cellular pathways and host immunity against invading viral pathogens than currently understood.

Previously we identified the MR as the sole anti-viral member of the nuclear receptor superfamily. Here, we investigate the effects of the MR on HSV-1 replication, and the mechanism of action, and show that the MC signalling pathway is anti-viral, that MR expression is upregulated in response to infection, and that this is dependent on both interferon (IFN) and interactions with the viral transactivator VP16 and cellular co-activator Oct1. Furthermore, the induction of IFN-β expression by the MR highlights its role in a feedback loop of regulation of innate immune defence mechanisms against HSV-1 infection.

## Results

### Inhibition of HSV-1 replication by the MR

**Figure 1:**
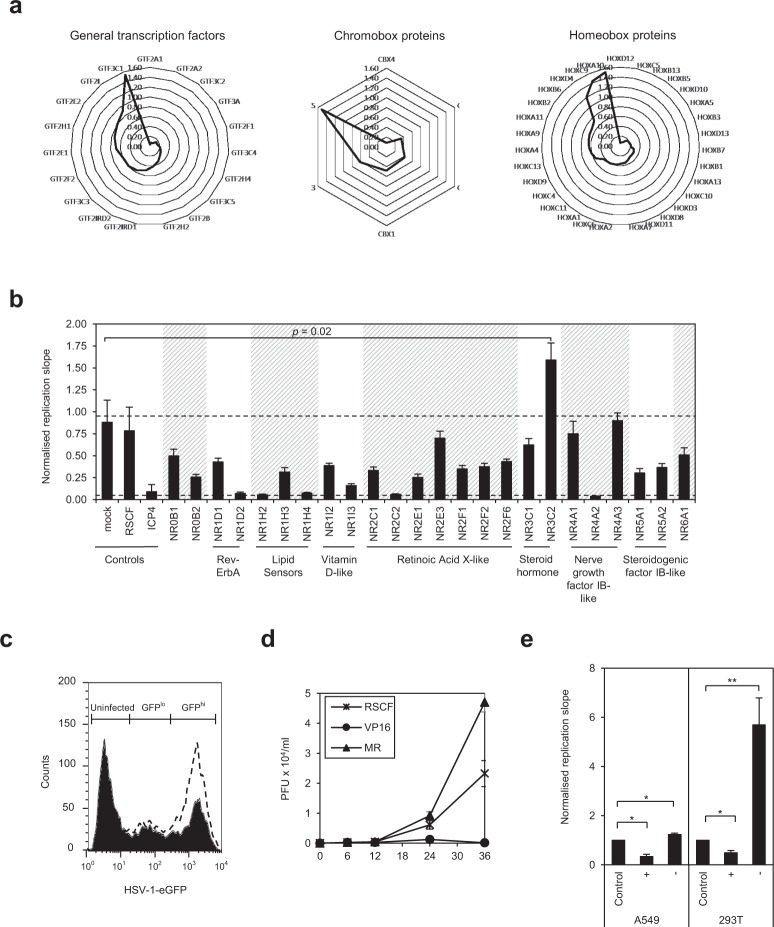
The MR (NR3C2) is anti-viral to HSV-1. (**a**) DNA-binding and transcription factors of the general transcription factor (GTF), chromobox (CBX) and homeobox (HOX) protein families affecting HSV-1 replication. HeLa cells were transfected with gene-specific siRNA before infecting with HSV-1-eGFP (MOI 0.5). Replication was monitored as a function of GFP fluorescence, and the slopes of replication during the linear phase were calculated, normalized to controls (average of mock and RSCF-transfected cells) and depicted by radar plots. (**b**) Depletion of the nuclear receptor NR3C2 enhances HSV-1 replication. HeLa cells were transfected with gene-specific siRNA and infected with HSV-1-eGFP (MOI 0.5). Replication was monitored and normalised as described. —cut-off for top 2.5% inhibitory (slope 0.05) and enhancing (slope 0.95) hits. Error bars represent the standard deviation of the mean of three independent experiments carried out in duplicates. *p-*value was calculated using an unpaired t-test for unequal variances. (**c**) Flow cytometric analysis of MR-depleted HSV-1-infected HeLa cells. Mock-transfected (solid line) or MR-specific siRNA-transfected (dashed line) HeLa cells were infected with HSV-1-eGFP (MOI 1) and GFP-positive cells quantified by flow cytometry 24 hr post-infection. (**d**) The MR is anti-viral to HSV-1. HeLa cells were transfected with negative control (RSCF), positive control (VP16) or MR-specific siRNA, and virus titre in supernatant harvested after 0, 6, 12, 24, and 36 hr post-infection was quantified by plaque assay on Vero cell monolayers. Graph represents an average virus quantity, in plaque-forming units (PFU) × 10^4^ per ml, from three experiments carried out in duplicate. (**e**) A549 or 293T cells overexpressing (+) or depleted for (−) MR were infected with HSV-1 (MOI 0.5) and replication monitored by GFP fluorescence. Slopes of replication over the linear phase were calculated and normalised to control (pCR3 or RSCF siRNA-transfected cells). Error bars represent the standard error of three independent experiments carried out in triplicates. *p*-values were calculated by unpaired two-tailed t-test for unequal variances. *p = 0.002; **p < 0.001.

We recently performed a gene depletion screen with a druggable genome siRNA library, targeting 7,237 human genes, to identify host factors affecting HSV-1 replication^5^. Analysis of this dataset for DNA-binding protein and transcription factor protein families found depletion of most members of the general transcription factor (GTF), chromobox (CBX), homeobox (HOX) (Fig. 1a) and nuclear receptor (NR) families (Fig. 1b), inhibited HSV-1. In all examples, however, depletion of one member led to an increase of virus replication. Nuclear receptors are a class of ligand-activated transcription factors which recognise thyroid and steroid hormones, and metabolites such as bile and fatty acids to directly activate gene expression. Of the 24 nuclear receptors within our siRNA library, all were pro-viral or had no effect, with the exception of the mineralocorticoid receptor (MR), NR3C2, whose depletion significantly enhanced virus replication (*p* = 0.02) (Fig. 1b). None of the siRNA pools were cytotoxic to HeLa cells following transfection (Table S1), confirming the phenotype of gene depletion on HSV-1 are specific and not due to effects on cell growth compromising virus replication.

In flow cytometric analysis of HSV-1-infected HeLa cells, MR depletion increased the proportion of HSV-1 infected cells from 53% in mock-transfected cells to ~80% (Fig. 1c). Furthermore, virus titre in cell supernatant was increased 2-fold following MR depletion by siRNA (Fig. 1d), confirming the MR is anti-viral to HSV-1 and effects are not due to changes in cell permissivity or GFP fluorescence in the absence of replication. We additionally looked at effects of the MR on HSV-1 replication in A549 and 293T cells, which have higher basal expression levels of the MR (www.proteinatlas.org)^19^. As anticipated, the anti-viral phenotype of the MR was reproducible, with MR overexpression inhibiting HSV-1 (~70% in A549 cells, and ~50% in 293T cells; *p*-value = 0.002) and MR depletion enhancing replication (~25% in A549 cells, and ~5 fold in 293T cells; *p*-values ≤ 0.002) (Fig. 1e). These data confirm that the inhibition of HSV-1 by the MR is a general and not cell-specific phenomenon.

### The MC signalling pathway is anti-viral to HSV-1

**Figure 2:**
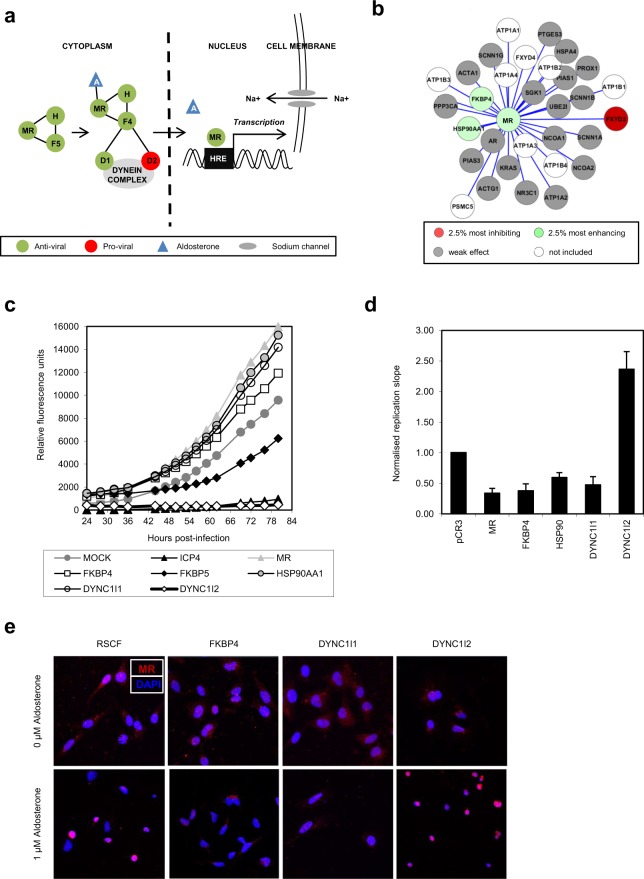
The MC signalling pathway is anti-viral to HSV-1. (**a**) The MC signalling pathway. Unliganded MR forms a cytoplasmic complex with the chaperone protein HSP90 (H) and the immunophilin FKBP5 (F5). Binding of the steroid hormone aldosterone (A) induces a structural change replacing FKBP5 with FKBP4 (F4), enabling nuclear translocation via the dynein complex and activation of transcription from the hormone response element (HRE) to regulate sodium reabsorption. (**b**) Protein-protein interaction networks of the MR. Human interaction information was compiled from public protein-protein interactions databases and curated pathways (KEGG and REACTOME) and combined with RNAi screen data for the top 2.5% most enhancing and most inhibiting genes (ranked by distance from the median replication slope). White nodes, not present in siRNA library; grey nodes, weak effect; red nodes, 2.5% most inhibiting; green nodes, 2.5% most enhancing. (**c**) Components of the MC signalling pathway are anti-viral to HSV-1. Virus growth curves in HSV-1-infected HeLa cells (MOI 0.5) depleted for members of the MC signalling pathway were plotted and compared to negative (mock-transfected) and positive controls (ICP4, an essential HSV-1 gene). (**d**) HSV-1 is inhibited by overexpression of MC signalling pathway members. 293T cells transiently over-expressing members of the MC signalling pathway were infected with HSV-1-eGFP at MOI 0.5 and replication monitored by GFP fluorescence. Replication slopes over the linear phase were calculated and normalised to controls (pCR3-transfected cells). Error bars represent the standard error of the mean of at least three independent experiments, carried out in triplicates. (**e**) The MR signalling complex is translocated to the nucleus via the dynein intermediate chain DYNC1I1. HeLa cells were transfected with positive control siRNA (FKBP4, essential for MR nuclear translocation), negative control siRNA (RISC-free, RSCF) or siRNA targeting the intermediate dynein chains DYNC1I1 or DYNC1I2 before transiently overexpressing HA-tagged MR and stimulating with aldosterone (1 μM). MR was detected with an anti-HA antibody (red) and nuclei visualised using a mounting medium containing DAPI (blue) by confocal microscopy.

The MR is a ligand-induced transcription factor, which in its unliganded state is located in a cytoplasmic complex with a host cell chaperone, HSP90, and the immunophilin, FKBP5 (FKBP51)^20,21^. Following diffusion of aldosterone into the cytoplasm, the hormone binds its receptor, induces conformational changes^22^ and replaces FKBP5 with FKBP4 (FKBP52)^23,24^, which in turn binds an intermediate chain of the dynein microtubule network to mediate nuclear translocation and activation of gene expression via the HRE^25,26^ (Fig. 2a). In addition to the MR, HSP90 and FKBP4 demonstrated a strong anti-viral phenotype in our siRNA screen (Fig. 2b). Furthermore, FKBP5, which binds an inactive form of the MR, was pro-viral, with depletion inhibiting HSV-1 by ~40% (replication slope 0.62 ± 0.13) (Fig. 2c). The dynein intermediate chain DYNC1I1 was anti-viral, similar to the MR (replication slope 1.23 ± 0.2), in comparison to DYNC1I2 which was significantly inhibitory (replication slope 0.02 ± 0.01) (Fig. 2c).

The HSV-1 replication phenotype following depletion of members of the MC signalling pathway was confirmed by siRNA deconvolution and replication assays in HeLa cells as used in the primary screen (Fig. S1a). Specific gene depletion was confirmed by RT-qPCR (Fig. S1b) and western blot (Fig. S1c). To further demonstrate the role of the MC signalling pathway in HSV-1 replication, transient gain-of-function assays were undertaken. Overexpression of the MR, FKBP4, HSP90 and DYNC1I1 inhibited HSV-1 replication (20%, 37%, 49% and 42%, respectively), whilst the pro-viral DYNC1I2 enhanced replication by ~2-fold (Fig. 2d). Such differences in inhibition may reflect variations in protein expression. Co-expression of multiple constituents of the MC pathway had a stronger effect on virus replication, with the MR, FKBP4, HSP90AA1 and DYNC1I1 together inhibiting HSV-1 by almost 75% (Fig. S1d).

DYNC1I1 displays the same phenotype as the MR and other members of the signalling pathway in siRNA gene depletion and protein overexpression studies, suggesting it is this dynein chain and not DYNC1I2 which mediates nuclear translocation of the MC signalling complex. Microscopic analysis of the sub-cellular localisation of these proteins in aldosterone-stimulated cells was undertaken to investigate this. Depletion of DYNC1I1 abrogated nuclear translocation of the MR, with a largely cytoplasmic distribution observed similar to removal of FKBP4, an essential mediator of nuclear translocation (Fig. 2e)^22^. In contrast, nuclear localisation of the MR was unaffected by depletion of DYNC1I2 and displayed a phenotype similar to control RSCF transfected cells. Combined, these data identify DYNC1I1 as the intermediate dynein chain responsible for nuclear translocation of the MC signalling complex, and suggest that a functional MC signalling pathway is required for inhibition of HSV-1 replication.

### Chemical inhibition of the MR enhances HSV-1 replication

**Figure 3:**
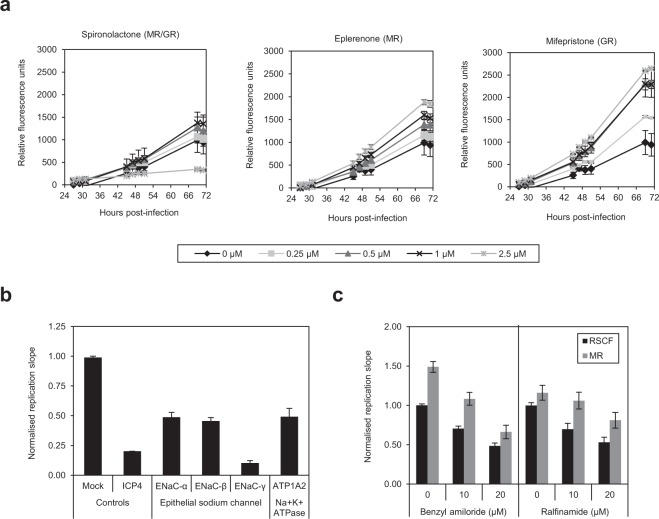
Chemical modulation of the MC pathway and sodium transport influences HSV-1 replication. (**a**) Modulation of HSV-1 replication by MR antagonists. The effect of MR antagonists on HSV-1 replication was determined by pre-treating HeLa cells for 24 hr with eplerenone, spironolactone or mifepristone at 0, 0.25, 0.5, 1 or 2.5 µM before infecting with HSV-1-eGFP (MOI 0.5) and measuring replication over multiple rounds. Virus replication curves are representative of three experiments carried out in triplicates, and error bars represent the standard error of the mean of technical replicates. (**b**) Sodium transport is required for HSV-1 replication in HeLa cells. ENaC subunits and the Na^+^K^+^ ATPase were depleted in the siRNA screen and HSV-1 replication monitored and normalised, as described. Error bars represent the standard error of three experiments done in triplicates. (**c**) The role of sodium transport in HSV-1 replication. The effect of blocking sodium ion transport via the epithelial sodium channel (ENaC; benzyl amiloride) or voltage-gated sodium channels (ralfinamide) was determined by treating control (RSCF) or MR siRNA-transfected and infected HeLa cells with increasing concentrations of inhibitor, measuring replication and comparing the calculated slopes of linear growth to untreated control cells. Error bars represent the standard error of the mean of three independent experiments done in triplicates.

As multiple components of the MC signalling network inhibit HSV-1, we investigated whether the anti-viral effects were linked and due to aberrant activation of the MC pathway. Treatment of HeLa cells with the MR-specific antagonist eplerenone mimicked siRNA depletion of the MR, and led to a dose-dependent increase in HSV-1 replication, with a peak of ~2.5-fold increase at the highest concentration (2.5 μM) (Fig. 3a). Spironolactone, the classical MR antagonist, enhanced replication by ~1.5-fold, a more moderate effect likely due to cross-reactivity with the pro-viral glucocorticoid (GC) signalling pathway. Mifepristone, a glucocorticoid receptor (GR)-specific antagonist, also enhanced HSV-1 in a dose-dependent manner (~2.5-fold at 2.5 μM), but as it can facilitate nuclear translocation of the GR this may be due to agonistic effects on the pro-viral GC pathway^27^. These data support our finding that the MC signalling pathway has anti-viral activity against HSV-1.

### Sodium influx is not involved in MR-induced inhibition of HSV-1 replication

A major function of the MR and downstream MC signalling is to induce a bi-phasic regulation of blood pressure via sodium reabsorption in response to aldosterone, via the apical epithelial sodium channel (ENaC; 30 mins post-stimulation) and the basal Na^+^K^+^ ATPase (>2.5 hr post-stimulation)^28^. If the MR exerts anti-viral effects by sodium transport it would be anticipated that blocking sodium transport would enhance virus replication. Depletion of the ENaC subunits α, β and γ, and the Na^+^K^+^ ATPase subunit 2, inhibited HSV-1 by at least 50% in comparison to control transfected cells (Fig. 3b), suggesting sodium transport is required for efficient HSV-1 replication. Treatment of HeLa cells with benzyl amiloride, an ENaC blocker, had a dose-dependent inhibitory effect on HSV-1 replication (Fig. 3c). As previously seen, MR depletion enhanced HSV-1 replication, even in the presence of increasing concentrations of benzyl amiloride. Ralfinamide, which blocks voltage-gated sodium channels, had the same effects on HSV-1 replication in the presence or absence of MR, albeit to lower levels. These data suggest that whilst sodium transport per se is required for HSV-1 replication, the MR and MC pathway inhibit HSV-1 via a mechanism independent of sodium transport.

### The MR affects transcription of viral genes at early stages post-infection

**Figure 4:**
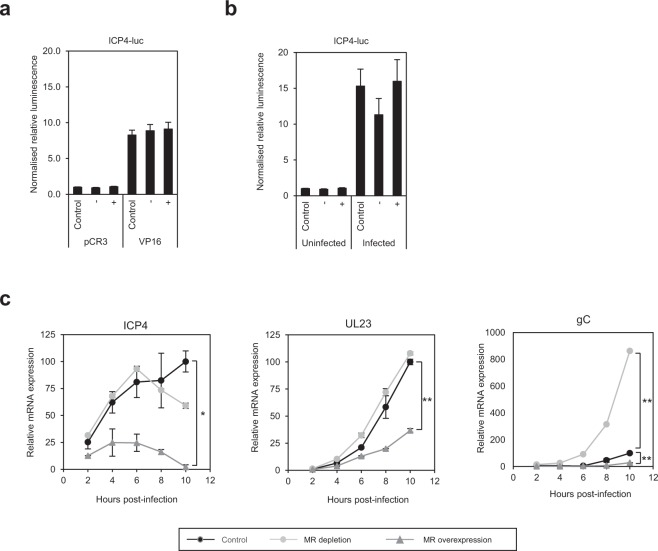
The MR affects transcription of viral genes at early stages post-infection. (**a**) The MR does not affect basal transcription of the ICP4 promoter. The MR was depleted (−) or overexpressed (+) in HeLa cells, before transfecting with control pCR3 or a pCR3-VP16 expression plasmid and an ICP4-luciferase plasmid. After 24 hr, luciferase activity was measured and normalised to controls (pCR3 plasmid alone). Error bars represent the standard error of at least three experiments carried out in triplicates. (**b**) The MR does not affect transcription of the ICP4 promoter during infection. The MR was depleted (−) or overexpressed (+) in HeLa cells before transfecting with an ICP4-luciferase plasmid. After 24 hr cells were infected with HSV-1 (MOI 1), luciferase activity measured after 8 hr and normalised to uninfected controls (RSCF siRNA or pCR3 plasmid). Error bars represent the standard error of at least three experiments carried out in triplicates. *p*-values for statistical significance were calculated by unpaired two-tailed t-test for unequal variances. *p ≤ 0.001. (**c**) The MR does not directly affect IE gene expression. The MR was depleted or overexpressed in HeLa cells before infecting with HSV-1 (MOI 1). RNA was extracted at 2, 4, 6, 8, and 10 hr post-infection and expression of HSV-1 genes from each temporal class (immediate-early, ICP4; early, UL23; late, gC) was quantified by RT-qPCR, normalised to the house-keeping gene HPRT and calibrated to control (RSCF siRNA or pCR3 transfected) cells at 2 hr post-infection. Error bars represent the standard error of duplicates and is representative of three independent experiments. *p*-values for statistical significance were calculated by unpaired two-tailed t-test for unequal variances. *p < 0.04; ** ≤ 0.001.

The MR and its pathway is anti-viral to HSV-1, and given its role as a transcription factor, the MR may influence HSV-1 by modulation of viral transcription. Effects of the MR on immediate-early (IE) gene expression was examined in luciferase assays with a reporter containing the ICP4 promoter. Neither overexpression nor depletion of the MR influenced basal transcription of the ICP4 promoter, and activation by the virion transcription factor VP16 was also unaffected (Fig. 4a). In context of infection with HSV-1, depletion of the MR led to a slight drop in activation of the ICP4-luc promoter, but this was not significant (Fig. 4b), suggesting the MR does not directly affect IE transcription.

Investigation into effects of the MR on the temporal cascade of viral transcription found expression of IE (ICP4), early (E; UL23) and late (L; gC) genes to be significantly repressed following overexpression of the MR (IE (*p* = 0.008), E (*p* = 0.0004) and L (*p* = 0.0008)) as early as 2 hr post-infection (Fig. 4c). Effects of MR depletion, however, were only evident during late viral gene expression, with levels of gC significantly enhanced (~8-fold) 10 hr post-infection (*p* = 0.001). A lack of phenotype following gene depletion may be indicative of redundancy in this transcriptional system, whilst inhibition by overexpression could be by direct repression of viral promoters, or by induction of anti-viral genes.

### The MR is upregulated by HSV-1 infection

**Figure 5:**
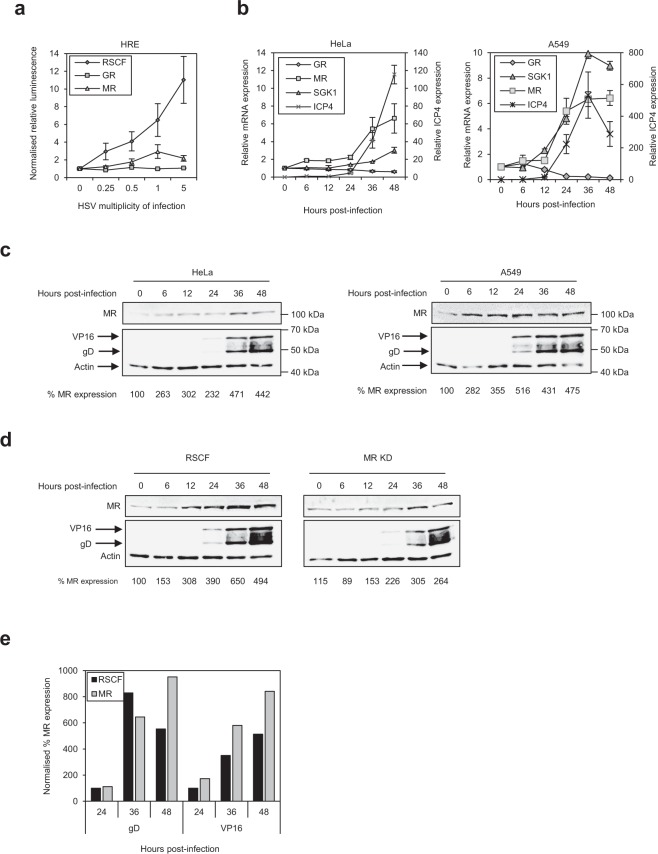
HSV-1 infection upregulates MR expression. (**a**) HSV-1 infection induces expression of the hormone response element (HRE). HeLa cells were transfected with an HRE-luciferase plasmid following siRNA depletion of the GR or MR, and activation in response to HSV-1 at increasing multiplicities of infection was quantified 24 h post-infection. Luciferase activity was normalised to uninfected, control RSCF-transfected cells. Error bars represent the standard error of four experiments done in triplicates. Raw data is provided in Table S4. (**b**) MC pathway genes are induced by HSV-1. HeLa or A549 cells were infected with HSV-1 (MOI 1) and expression of ICP4 (right-hand axis), GR, MR and SGK1 (left-hand axis) quantified by qPCR, normalised to HPRT and calibrated to uninfected (GR, MR and SGK1) or 3 h post-infection (ICP4) cells. Error bars represent the standard error of four experiments done in duplicate. (**c**) HSV-1 infection increases MR protein expression. HeLa or A549 cells were infected with HSV-1 (MOI 1), and the MR, and viral proteins VP16 and gD, detected by western blot at 0, 6, 12, 24, 36, and 48 h post-infection. (**d**) Induction of MR protein by HSV-1 following MR depletion. HeLa cells were transfected with control RSCF or MR-specific siRNA before infecting with HSV-1 (MOI 1) and detecting MR and viral proteins VP16 and gD by western blot at a range of times post-infection. (**e**) Depletion of MR enhances viral protein expression. HeLa cells transfected with control RSCF or MR-specific siRNA were infected with HSV-1 (MOI1) before samples harvested at a range of times, and viral proteins gD and VP16 detected by western blot. MR and viral protein expression (Panels C and D) were normalised to actin and expressed as % uninfected cells (24 h p.i. for viral proteins). Images were detected by Licor, and bands quantified in ImageStudio. Panel c linear signal ranges were 94,735–114,000 (HeLa, actin), 6,090–28,700 (HeLa, MR), 54,000–86,600 (A549, actin), and 7,710–52,000 (A549, MR). Panel d linear signal ranges were 81,110–165,923 (actin), 11,731–93,328 (MR), 36,080–185,743 (VP16), and 29,929–153,643 (gD) (Panel d). Data from western blots are representative of three experiments carried out in duplicates. Images have been cropped. Full gels are shown in Fig. S4.

Effects of the MR on viral gene expression, and thus HSV-1 replication, at early times post-infection may be due to induction of anti-viral genes in response to infection. The MR, and the GR, induce expression of genes via the hormone-response element (HRE), and at composite response elements with other transcription factors, following ligand binding (S2a)^29,30^. The ability of HSV-1 to induce genes via the HRE was thus investigated. Transcription from an HRE-luciferase reporter was activated by HSV-1, in a dose-dependent manner, with ~10-fold increase in activity observed at the highest MOI (MOI 5) (Fig. 5a).

As the MR and SGK1 promoters both contain an HRE^31–33^, we tested whether HSV-1 infection upregulates their expression. As determined by qPCR analysis, expression of the pro-viral GR was downregulated in both HeLa and A549 cells (Fig. 5b). This down-regulation may represent a genuine repression of pro-viral genes in response to infection, or be a symptom of host-cell shutoff induced by HSV-1 infection^34^. In contrast, MR expression was upregulated ~5–6-fold post-infection in both cell types at the gene and protein level (Fig. 5c). Additionally, expression of SGK1 in HeLa cells was moderately increased (~3-fold) at the gene and protein level by HSV-1 infection (Figs 5b and S2b). Induction of SGK1 mRNA expression in A549 cells was greater still, reaching ~10-fold increase at 36 h post-infection (Fig. 5b).

Effects of HSV-1 infection on MR protein expression following MR depletion was also analysed. MR expression in control siRNA (RSCF)-transfected cells correlated with that seen in untransfected, infected cells, with peak expression (~6.5-fold increase) occurring at 36 h post-infection (Fig. 5d). As expected, whilst the MR followed the same overall pattern of expression in MR-depleted cells as that seen in RSCF-transfected cells (increasing over time to 36 h p.i.), levels were consistently ~50% lower at each time point (Figs 5d and S2c). Protein expression of SGK1 followed the same trend as seen with the MR, where expression increased during infection, but overall levels were lower in cells depleted for the MR (Fig. S2d). Interestingly, quantification of viral proteins found expression of both gD and VP16 to be enhanced following MR depletion, at all time-points (VP16) or by 48 h post-infection (gD) (Fig. 5e), providing further evidence for the anti-viral phenotype of MR against HSV-1.

As SGK1, the major transcriptional target of the MR, along with other targets including NDRG2 and EGFR, were identified in our screen as pro-viral (Fig. S2e), these data suggest either an induction of anti-viral (MR) steroid hormone receptors by the host cell as part of its defence against HSV-1, or a regulation of MR expression, and thus its downstream targets, by a viral transcription factor.

### The MR, VP16 and its cellular co-regulator Oct1 synergistically activate HRE promoters

**Figure 6:**
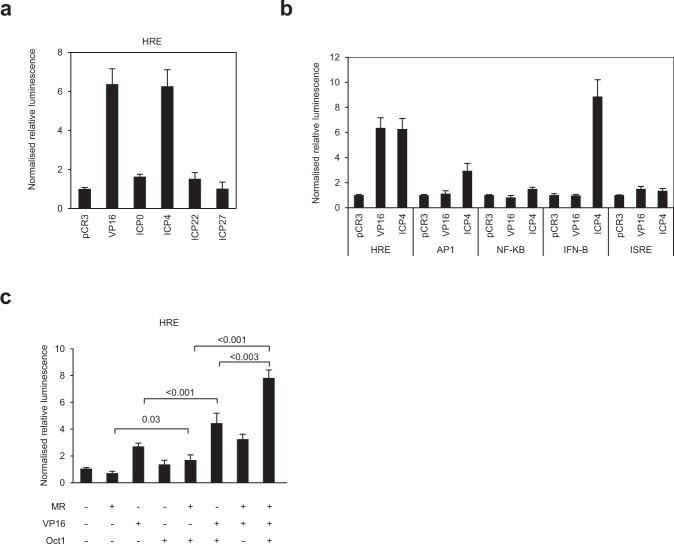
The MR, VP16 and its cellular co-regulator Oct1 synergistically activate HRE promoters. (**a**) VP16 and the immediate-early (IE) protein ICP4 activate the HRE. VP16 and HSV-1 IE proteins were overexpressed in HeLa cells with the HRE-luc reporter, and luciferase activity was measured and normalised to control transfected cells (pCR3) after 24 hr. Error bars represent the standard error of the mean of three independent experiments carried out in triplicates. Raw data is provided in Table S5. (**b**) VP16 specifically activates the HRE promoter element. VP16 was overexpressed in HeLa cells with HRE, AP-1, NF-κB, IFN-β or ISRE reporters, and luciferase activity measured and normalised to control transfected cells (pCR3) after 24 hr. Error bars represent the standard error of the mean of three independent experiments carried out in triplicates. Raw data is provided in Table S6. (**c**) The MR, VP16 and Oct1 synergistically transactivate expression from the HRE. The MR, VP16 and Oct1 were transfected into HeLa cells alone or in combinations with the HRE-luciferase reporter. After 24 hr, luciferase activity was measured and normalised to control transfected cells (pCR3). Error bars represent the standard error of the mean of at least three independent experiments carried out in triplicates. *p-*values were calculated by unpaired two-tailed t-test for unequal variances. Raw data is provided in Table S7.

During infection, the virion-incorporated transcription factor VP16 initiates a temporal cascade of viral gene expression via the immediate-early genes ICP0, ICP4, ICP22, ICP27 and ICP47, which, with the exception of ICP47, act as transcription factors to regulate later stages of viral gene expression We investigated whether these viral transcription factors could activate the HRE and thus MR expression during HSV-1 infection. Both VP16 and ICP4 activated the HRE ~6-fold (Fig. 6a), and whilst ICP4 additionally activated cellular promoters (AP1 and IFN-β, 6- and 8-fold, respectively), VP16 was specific in its activation of the HRE (Fig. 6b). Activation was enhanced to some degree by co-expression with the MR (Fig. S3a) and reduced by siRNA depletion of the MR (Fig. S3b). Whilst this did not reach significance, the lack of the Octamer-VP16-binding element in the HRE reporter construct suggests VP16 may modulate MR-mediated activation of HRE-containing transcripts.

In luciferase reporter assays with MR, VP16, and Oct1, its cellular interaction partner and transcriptional co-regulator, co-expression of Oct1 with either the MR or VP16 almost doubled HRE activation from that seen with the MR or VP16 alone (*p* = 0.03 or < 0.01, respectively; Fig. 6c), although Oct1 alone activated similarly to Oct1 plus MR. The synergistic effect of these components was significantly more pronounced (~8-fold activation) when all three proteins were expressed together. In contrast, overexpression of VP16, MR and Oct1 only slightly, but significantly, increased transactivation of the viral ICP4 promoter, in comparison to VP16 alone (*p* = 0.008; Fig. S3c). These data suggest that the viral transactivator VP16 acts in concert with its partner Oct1 to enhance MR-mediated activation of HRE-containing genes during infection.

### The anti-viral phenotype of the MR involves interferon

**Figure 7:**
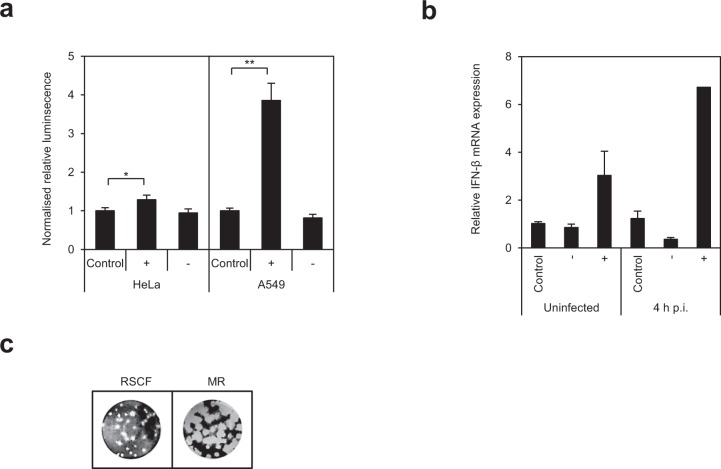
The anti-viral phenotype of the MR involves interferon. (**a**) The MR induces a soluble factor able to activate ISRE promoter elements. Supernatant from HeLa or A549 overexpressing (+) or depleted for (−) the MR was transferred to 293T cells expressing ISRE-luc reporter plasmid, and ISRE activation quantified after 24 hr by lysing cells and measuring luciferase activity. Activation was normalised to controls (RSCF siRNA or pCR3 plasmid). The experiment was carried out twice with six replicates, and error bars represent the standard error of the mean of all replicates. *p-*values were calculated by unpaired two-tailed t-test for unequal variances. *<0.05; **<0.001. (**e**) The MR enhances IFN-β expression in response to HSV-1. The MR was depleted (−) or overexpressed (+) in HeLa cells before infecting with HSV-1 (MOI 1) and harvesting RNA at 0, and 4 h post-infection (p.i.). IFN-β was quantified by qPCR, and normalised to control transfected, uninfected cells. (**f**) MR depletion increases viral plaque number and size. Supernatant from control or MR siRNA-transfected, HSV-1-infected HeLa cells (MOI1) was harvested after 24 h, and used to infect Vero cells. Cells were fixed and stained after 72 hr.

Given our observations that the MR inhibits viral gene expression upon infection, and that anti-viral (MR) steroid hormone receptors are induced during infection, we investigated whether these may form part of the interferon-mediated host cell defence mechanism against HSV-1. The capacity of supernatant from HeLa and A549 cells either depleted for or overexpressing the MR to activate an interferon-responsive ISRE-luc reporter was determined. Whilst supernatant from HeLa cells depleted for the MR caused only minimal changes in ISRE-luc activity, MR overexpression increased activation by 30%, narrowly missing statistical significance (Fig. 7a; *p*-value = 0.06). In A549 cells, however, effects were much more striking, with depletion reducing ISRE activation by 20% and overexpression leading to an almost 4-fold increase in ISRE activation (Fig. 7a; *p*-value < 0.0001). The relatively moderate effects of MR depletion are likely due to the lack of external stimulation in these cells.

As the ISRE is activated by IFN-β, the ability of the MR to affect expression of IFN-β in response to HSV-1 infection was determined. Overexpression of the MR led to ~3-fold increase in basal expression of IFN-β (Fig. 7b), an effect that was more pronounced at 4 h post-infection (~7-fold increase). Again, MR depletion was less significant, with no effect in uninfected cells, likely due to the absence of stimulation, and ~3-fold inhibition of IFN-β expression at 4 hr post-infection (*p*-value = 0.02). Accordingly, analysis of plaque formation in Vero cells infected with supernatant from MR-depleted and HSV-1-infected cells found both plaque number and size to be increased, indicative of decreased levels of interferon (Figs 1d and 7c).

Together, these data show that MR expression is induced in response to HSV-1 infection, and suggest the MR may inhibit HSV-1 replication by multiple mechanisms: by direct repression of viral promoters, by induction of potentially anti-viral HRE-containing cellular genes, and by induction of interferon in response to infection. Further investigations into the role of the MR in interferon induction will provide additional insight into these novel roles of the MR in the control of viral infection.

## Discussion

We previously identified the mineralocorticoid receptor (MR) as the sole anti-viral member of the steroid hormone receptor family^5^. As a ligand-activated transcription factor, the MR primarily acts on polarised epithelial cells to regulate sodium reabsorption and thus blood pressure in response to its ligand aldosterone. However, the MR is broadly expressed and has recently been shown to have additional functions outside the kidney. In this paper we have investigated the mechanism by which the MR inhibits HSV-1 replication.

The MR exists in a cytoplasmic complex with the host chaperone protein HSP90 and the immunophilin FKBP5 which, following binding of the ligand aldosterone, is replaced with FKBP4 to mediate nuclear translocation via the dynein intermediate chain network. We found both HSP90 and FKBP4 to be anti-viral, whilst FKBP5 was required for virus growth. Our previous work noted the importance of the dynein motor complex for nuclear transport of the HSV-1 capsid^5^, and assumed disparate phenotypes between the intermediate chain DYNC1I1 (depletion enhanced virus growth) and other components of the motor complex (depletion inhibited virus growth) to be a consequence of functional redundancy. Combined with siRNA depletion data of the MC pathway, and observations that MR antagonists reproduced the phenotype of MR depletion, these data suggest the MC signalling pathway, as opposed to its individual components, is anti-viral to HSV-1, and that transcriptional activity of the MR is essential for its inhibition of HSV-1 replication. The reproducibility of this anti-viral phenotype in a range of cell types with varying basal expression levels of the MR confirms this to be a general effect of the MR, and not restricted to our siRNA screen experimental conditions and cell type.

The MR primarily regulates blood pressure in response to aldosterone by modulating genes involved in salt (Na^+^ and K^+^) transport, yet, perhaps not surprisingly, we found effects of the MC pathway on HSV-1 to be independent of sodium transport. Investigations into regulation of viral promoters by the MR found activity of an ICP4-luciferase promoter was unaffected by both overexpression and depletion of the MR, either alone, after activation by its viral transactivator VP16, or during infection. Expression of all temporal classes of viral genes (immediate early, early and late genes) during infection was, however, suppressed by MR overexpression as early as 2 hr post-infection, whilst only late gene expression was enhanced by MR depletion. The MR modulates gene expression by direct binding to hormone-response elements (HRE), or the forming heterodimers with other transcription factors, with additional specificity and complexity, as well as determinants of gene activation or inhibition, provided by surrounding sequences (reviewed in^29^). MR overexpression may therefore have direct inhibitory effects on viral gene expression.

The differing phenotypes of MR overexpression and depletion on IE, E, and L viral gene expression could be due to two things. Firstly, by functional redundancy in this transcriptional system. IE and E gene expression is dependent upon viral transcription factors alone (IE genes) or in combination with cellular factors (E genes), whilst L gene expression is dependent upon a preinitiation complex of cellular proteins binding the TATA box and transcriptional start site (Inr), often stabilised by upstream cellular transcription factors or downstream activation sequences (DAS)^35^. As IE and E genes are predominantly regulated by viral genes, depletion effects may only be apparent during L gene expression due to their greater reliance upon cellular factors.

Secondly, the MR or downstream targets may induce expression of anti-viral genes, which, if induced following MR transfection, 24 hr in advance of infection, may prime cells in an anti-viral state and render incoming virus less infectious. Depletion effects would be observed only at later stages more consistent with inhibition of replication by host immune responses. A role has recently been identified for the MR-regulated gene SGK1 in IFN-γ expression and anti-viral immune responses^18^. Furthermore, the closely related glucocorticoid receptor (GR) mediates glucocorticoid-dependent suppression of anti-viral type I IFN immune responses^11,36^. Here, we found overexpression of the MR to enhance basal expression of IFN-β mRNA, an effect which was more pronounced at early (4 hr) time-points post-infection, and MR depletion to reduce IFN-β mRNA expression, as well as increase both viral plaque number and size. In addition, supernatant from cells overexpressing the MR activated an IFN-β-responsive ISRE-luc reporter. These data support a role for host immunity in the phenotype of MR overexpression and depletion on viral gene transcription.

As observed by others, we found the HRE to be activated by HSV-1 infection (Fig. 5a)^37^, and additionally identified a corresponding increase in expression of both the MR and its target SGK1. MR expression is self-regulated via several HRE half-sites in the P2 promoter region and an inverted HRE-like sequence in the P1 promoter region^33,38^, but further analysis of these promoter regions (http://www.ifti.org/Tfsitescan/) identified IFN-α, IRF2 and ISRE-responsive elements. Given our observations that the MR enhances expression of IFN-β in response to HSV-1 infection, regulation of its own expression by the host immune responses, and thus the existence of a feedback loop of interferon and MR expression, is an interesting avenue for future work.

Two HSV-1 immediate-early proteins activated expression of the HRE – ICP4 and VP16 – and whilst ICP4 was relatively promiscuous, VP16 was specific in its activation of the HRE-luciferase reporter. VP16 induces viral IE gene expression via the TAATGARAT and GCGGAA *cis*-regulatory elements by recruitment of the cellular co-regulator Oct1^39^. These motifs are absent in the HRE, and the other *cis*-regulatory element luciferase reporters tested here, but as transcriptional activation of viral IE promoters is provided by Oct1^40^, VP16 may modulate HRE upon infection by recruiting or modulating the activity of HRE-specific cellular transcription factors. We found Oct1 and the MR acted synergistically to enhance VP16-mediated upregulation of the HRE, in a manner similar to the known synergistic activation of the MMTV LTR by GC and Oct1/Oct2^41^. Furthermore, in co-immunoprecipitation experiments, the MR interacted with both Oct1 and VP16, in overexpression experiments and during infection (data not shown).

The MR recruits co-regulators via the N-terminal activation function (AF)−1 region, or the AF-2 region within the C-terminal ligand-binding domain (reviewed^42^), and the consensus L-X-X-L-L motif for co-activator binding to AF-2^43–45^ is present in the DNA-binding POU_S_ domain of Oct1^46^. Moreover, analysis of the amino acid sequence of the cellular protein HCF-1, required to stabilise the VP16:Oct1 complex on viral IE promoters^47^, found it also contains this motif (data not shown). We hypothesise this could help stabilise MR-containing transcription complexes. Analysis of the MR promoters identified an ICP4-binding motif (ATCGTC) in P1, which may further contribute to the regulation of MR expression following HSV-1 infection^48^.

We propose that during infection, VP16 in incoming viral particles forms a complex with Oct1 and MR, and localises to MR- and Oct1-responsive promoters during the early stages of infection. This leads to an induction of MR and downstream targets such as SGK1, which has two roles: (1) potential repression of IE, E and L viral gene expression, and (2) induction of IFN-β expression. Future work will focus on confirming MR:Oct1 and MR:VP16 interactions during infection, identifying potential MR-interaction sequence(s) within VP16, further investigating the formation of a MR:VP16:Oct1 complex on HRE-containing promoters, and elucidating the role and mechanisms of action of the MR in innate immunity.

## Materials and Methods

### siRNA screen and validation by SMARTpool deconvolution

The siRNA screen was carried out as previously described^5^. Briefly, HeLa cells were reverse-transfected in triplicate with individual siRNA SMARTpools (4 siRNAs per gene; sequences shown in Table S2) at 50 nM using 0.1% Dharmafect 1 (DF1; Dharmacon, ThermoFisher Scientific). One replicate was used to determine cytotoxicity of gene depletion (CellTiter Blue; Promega) (Table S1) whilst duplicate plates were infected with HSV-1-eGFP strain C12^49^ at a multiplicity of infection (MOI) of 0.5, and replication quantified over multiple rounds [20–80 hr post-infection (p.i.)] by monitoring GFP fluorescence. Correlation of viral titre (in plaque-forming units; PFU) to GFP fluorescence shows this to be a valid method of quantifying viral replication (Fig. S7a), and western blot detection of viral proteins in HSV-1-eGFP C12-infected HeLa cells (Fig. S7b) confirms viral replication equates to increases in GFP fluorescence. Replication slopes over the linear phase were calculated and normalized to mock transfected wells on individual assay plates, and the mean replication slope of six replicates used for subsequent data analyses. Primary screen phenotypes were confirmed by deconvolution of siRNA SMARTpools (four siRNAs per gene tested individually), and considered validated if the phenotype was reproducible by two or more of the four siRNAs.

### Quantification of viral titre by plaque assay

For gene depletion, 5 × 10^4^ HeLa cells were reverse-transfected with 50 nM siRNA using 0.15% DF1 in 48-well plates, and incubated for 48 hr. For cDNA overexpression, 5 × 10^4^ HeLa cells were seeded in 48-well plates before transfecting with 250 ng control pCR3 or pCR3-MR DNA with 0.2% Lipofectamine 2000 (Invitrogen). Cells were infected with HSV-1 (MOI 1) and supernatant harvested at 6, 12, 24, 36, or 48 hr p.i. For virus quantification, 1 × 10^5^ cells Vero cells were seeded in 24-well plates before infecting with 150 μl virus supernatant for 1 hr at 37 °C. Inoculum was removed and cells overlaid with 1 ml DMEM/5% FCS with 0.8% agarose before fixing with 1% formaldehyde, removing agarose plugs and staining cells with crystal violet after 72 hr. Plaques were counted and titres calculated as PFU per ml.

### Bioinformatic analyses

Interactions between HSV-1 and human proteins were identified by text mining using Syngrep^50^, and all hits manually curated^51^. Host-virus interactions were connected to a network of 62,310 human protein-protein interactions assembled from HPRD^52^, DIP^53^, BIND^54,55^, INTACT^56^, MINT^57^, and BIOGRID^58,59^. Data on known human protein complexes was retrieved from the CORUM database, and complexes with subunits showing consistently stronger effects (inhibiting or enhancing) than expected by chance were detected using Wilcoxon’s rank-sum test. Genes included in the RNAi screen were ranked by distance from the median knockdown, with the most inhibiting and enhancing genes being ranked highest. False discovery rate (FDR) was used for multiple testing correction^60^.

### Flow cytometry

2 × 10^5^ HeLa cells were transfected in 12-well plates with 50 nM siRNA using 0.15% DF1, and, after 48 hr, infected with HSV-1-eGFP (MOI1) for 1 hr at 37 °C. Inoculum was removed, replaced with 2 ml growth medium, and cells harvested 48 hr p.i. Trypsinised cells were washed in phosphate buffered saline (PBS), pelleted by centrifugation for 10 min at 199 *g* and fixed in 4% paraformaldehyde before analysing eGFP expression by flow cytometry (FACS DiVa, BD Biosciences; CellQuest software).

### Microscopic analysis of MR nuclear translocation

siRNA (RSCF, FKBP4, DYNC1I1 or DYNC1I2) was reverse-transfected into 3 × 10^4^ HeLa cells at 50 nM with 0.15% DF1 in 8-well chamber slides (Becton Dickinson), and the next day transfected with 350 ng MR in a C-terminal HA-tagged pCR3 expression vector with 0.2% Lipofectamine 2000. The next day, cells were serum-starved for 24 hr before incubating on ice for 1 hr with serum-free DMEM or 1 µM Aldosterone (diluted in serum-free DMEM) and inducing nuclear translocation by incubation at 37 °C for 15 mins. Cells were rinsed in PBS, fixed in 1% formaldehyde and autofluorescence quenched with 50 mM NH_4_Cl for 10 minutes before permeabilisation in 0.5% Triton and staining with anti-HA (Roche, UK; 1:200) or IgG2a isotype control antibody (1:500) in PBS/1% bovine serum albumin (BSA). Proteins were detected with a goat anti-rat Alexa594-conjugated antibody (Invitrogen; 1:500). Slides were mounted in anti-fade medium containing DAPI and images acquired by confocal microscopy with a 40x oil-immersion objective (Zeiss LSM710; Zen 2009 software).

### RT-qPCR

#### Confirmation of siRNA gene depletion

HeLa cells were reverse-transfected in triplicate with 50 nM siRNA and 0.15% DF1, and RNA harvested after 48 hr in 100 µl TRIzol (Invitrogen). Triplicate wells were combined, RNA extracted by phenol:chloroform, and mRNA quantified by TaqMan qPCR (Verso One-step RT-qPCR kit, Thermofisher) or SYBRgreen qPCR (Thermofisher; denoted by *) with gene-specific primers and probes, where possible (Roche Universal Probe Library), or by one-step SYBRgreen RT-qPCR kit (Thermofisher)(Table S3). Briefly, 20 ng RNA was added to white qPCR plates in duplicates for each primer-pair, and genes amplified in a 10 µl reaction with 500 nM forward and reverse primers, and 125 nM probe. In this and all RT-qPCR assays, mRNA expression was normalized to the housekeeping cellular gene hypoxanthine phosphoribosyltransferase 1 (HPRT), calibrated to mock-transfected cells, and the mean expression calculated.

#### siRNA gene depletion and HSV-1 infection time-course

Cells were transfected as described above in plaque assay methods, and infected with HSV-1-eGFP C12 (MOI 0.5) after 48 hr before cells were harvested and RNA extracted as described above at 0, 2, 4, 6, 8, and 10 hr p.i. mRNA levels of viral (ICP4, UL23, gC) or cellular genes (GR, MR, SGK1) were determined by TaqMan or SYBR green RT-qPCR as described.

#### HSV-1-mediated gene induction

HeLa or A549 cells were seeded in 12-well plates at 2 × 10^5^ cells per well, and the next day infected with HSV-1 (strain C12) at MOI1. Cells were rinsed in PBS and lysed in 300 µl TRIzol after 0, 6, 12, and 24 hr p.i. before RNA was extracted and RT-qPCR carried out as described above.

### HSV-1 replication assays

#### Transient cDNA overexpression and HSV-1 replication assays

HeLa or A549 cells were seeded at 1 × 10^4^ cells/well in black 96-well plates and the following day transfected with 37.5 ng of DNA from components of the MC signalling pathway, alone or in combination, made up to a total of 150 ng DNA with pCR3 using Lipofectamine 2000 at 0.2%. Cells were incubated for 24 hr before infecting with HSV-1-eGFP (Strain C12) at MOI 0.5. After 1 hr, inoculum was removed, replaced with phenol red-free growth media and replication growth curves were monitored. The slope over the linear phase of replication was calculated and normalized to pCR3-transfected cells.

#### siRNA depletion and HSV-1 replication in other cell types

1 × 10^4^ A549 cells, or 2 × 10^4^ 293T cells were seeded in black 96-well plates with 50 nM negative control (RSCF), positive control (ICP4; 293T cells) or MR siRNA, with 0.15% DF1 and after 24 hr, were infected with HSV-1-eGFP (MOI 0.5). After 1 hr inoculum was removed and replaced with phenol red-free growth media and replication growth curves were monitored. The slope over the linear phase of replication was calculated and normalized to RSCF control cells.

#### Mineralocorticoid/glucocorticoid antagonist assays

HeLa cells were seeded in black 96-well plates at 1 × 10^4^ cells/well and the following day, were pre-incubated with mifepristone, eplerenone, or spironolactone dissolved in ethanol and diluted to 0.25, 0.5, 1 or 2.5 µM in phenol red-free DMEM. After 24 hr, cells were infected with HSV-1-eGFP (MOI 0.5) and replication in the presence of antagonists was measured over multiple rounds of replication by monitoring GFP fluorescence.

#### Sodium ion channel inhibitor assays

1 × 10^4^ HeLa cells were reverse-transfected in black 96-well plates with 50 nM RSCF or MR siRNA, with 0.15% DF1 and after 24 hr, were infected with HSV-1-eGFP (MOI 0.5). After 1 hr inoculum was removed and replaced with benzyl amiloride or ralfinamide, dissolved in ethanol and diluted to 0, 10 or 20 µM in phenol red-free media. Replication was monitored as GFP fluorescence over multiple rounds, and replication slopes over the linear phase normalised to untreated, RSCF-transfected cells.

### Luciferase reporter assays

#### Activation of HRE by mineralocorticoid and glucocorticoid ligands

1 × 10^4^ HeLa cells were seeded in 96-well plates and the following day transfected in triplicates with 30 ng HRE-luc and 70 ng control pCR3 expression plasmid. After 24 hr cells were serum-starved for 8 hr (aldosterone treatment) before stimulating with the synthetic glucocorticoid dexamethasone (1 μM) or the mineralocorticoid aldosterone (5 μM) for a further 24 hr. Cells were lysed in 30 µl Cell Culture Lysis Reagent (CCLR, Promega) before 30 µl luciferase substrate (Promega) was added and luciferase activity quantified by measuring luminescence and normalising to an average activity in unstimulated cells.

#### Activation of HRE-luc by HSV-1

1 × 10^4^ HeLa cells were reverse-transfected with 50 nM RSCF, GR, or MR siRNA and 0.15% DF1 and the next day transfected with 40 ng HRE-luc reporter plasmid^61^ and 60 ng pCR3 expression plasmid with 0.2% Lipofectamine 2000. After 24 hr, cells were infected with HSV-1-eGFP at a range of multiplicities, and luciferase activity at 24 hr p.i. quantified as above and normalised uninfected cells for each siRNA treatment.

#### Gene depletion and overexpression assays

1 × 10^4^ HeLa cells were reverse-transfected with RSCF, MR or VP16 siRNA (50 nM) using DF1 (0.15%) and the next day cells were transfected with 40 ng HRE- or ICP4-luc, and 60 ng control pCR3 expression plasmid, pCR3-MR or pCR3-VP16 with 0.2% Lipofectamine 2000. Cells were lysed after 24 hr and luciferase activity monitored as described. For overexpression, cells were seeded and the next day transfected with 30 ng ICP4- or HRE-luc, and 60 ng empty pCR3 plasmid or pCR3-VP16, with 0.2% Lipofectamine 2000. Cells were lysed after 24 hr, and luciferase activity monitored as described. For gene depletion or overexpression combined with HSV-1 infection, HeLa cells were seeded and transfected with siRNA and pCR3 plasmid as described above. 24 hr after plasmid overexpression cells were infected with HSV-1-eGFP at MOI 1 and lysed after 8 hr. Luciferase activity was quantified as described.

#### Activation of promoter elements by HSV-1 transcription factors

1 × 10^4^ HeLa cells were seeded in 96-well plates and the next day transfected with 30 ng HRE, NF-KB, AP1, ISRE, IFN-β or ICP4-luc, and 60 ng control pCR3 or pCR3-VP16, -ICP22, -ICP27, or pEYFP-ICP4, with 0.2% Lipofectamine 2000. Cells were lysed after 24 hr and luciferase activity quantified as described. The enhancer element sequences of luciferase reporters are: HRE-luc (GGTACATTTTGTTCTAGAACAAAATGTACC)_2_; NF-KB-luc (T**GGGGACTTTCC**GC)_5_; AP1-luc (**TGACTAA**)_7_; ISRE-luc (T**AGTTTCACTTTC**CC)_5_. The IFN-β-luc reporter (p125-luc) contains the promoter region (−125 to + 19) of human IFN-β upstream of the luciferase gene in pGL3-basic. The ICP4-luc reporter contains a 361 bp region of the ICP4 promoter^61^.

#### Transient co-expression of MR, VP16 and Oct1

1 × 10^4^ HeLa cells were seeded the day before transfecting with 30 ng ICP4-luc or HRE-luc and 40 ng of each expression plasmid using 0.2% Lipofectamine 2000. For single-gene transfections (MR, Oct1, VP16 alone), DNA quantity was equalised using control pCR3 expression plasmid. After 24 hr, cells were lysed and luciferase activity monitored as described.

#### Activation of ISRE-luc by supernatant

5 × 10^4^ HeLa or A549 cells were reverse transfected in 48-well plates with 50 nM siRNA (control RSCF or MR) and 0.15% DF1, or forward-transfected with 500 ng pCR3 or pCR3-MR DNA and 0.15% Lipofectamine 2000. After 48 hr (siRNA transfection) or 24 hr (cDNA overexpression), supernatant was harvested and frozen. 293T cells were seeded at 3 × 10^4^ cells/well in 96-well plates, and the next day transfected with 30 ng ISRE-luc and 0.15% Lipofectamine 2000. After 24 h, cells were lysed and luciferase activity monitored as described above.

### Western blot analysis of protein expression

#### Time-course infection

HeLa or A549 cells were seeded in 24-well plates at 1 × 10^5^ cells/well and the next day infected with HSV-1 C12 (MOI1). Cells harvested at 0, 6, 12, 24, 36, and 48 p.i. were rinsed in PBS, lysed in 100 μl 2x Laemmli sample buffer and boiled for 10 min before separation on a 12% polyacrylamide gel and transfer to nitrocellulose membrane. After blocking in 5% milk/TBS-tween, membranes were incubated with anti-β-actin (Cell Signalling, clone 8H10D10; 1:3000), MR (Abcam [ab62352]; 1:500; detects ~90 kDa band), or viral VP16 (Abcam; 1:100; detects ~56 kDa band)^62^, gD^63^ and gB^64^ (hybridoma supernatant, 1:100) antibodies, followed by secondary incubation with HRP-conjugated anti-mouse (actin, viral proteins; 1:10,000) or anti-rabbit (MR, 1:5000) antibody. Proteins were detected with ECL Western blotting detection system and quantified in ImageStudio after Licor imaging. Within each Figure, samples were derived from the same experiment and run on the same gel. When necessary, transferred membranes were cut, and stained and imaged independently. When quantified, bands were normalised to actin.

#### Gene depletion

1 × 10^5^ HeLa cells were transfected in 24-well plates with RSCF, VP16, MR or GR siRNA (50 nM) using DF1 (0.15%), and after 48 hr, cells were infected with HSV-1 C12 (MOI 1), harvested at a range of times, and proteins detected and quantified as described above.

## Electronic supplementary material

Supplementary Information
