# Supplementary material for: Antiviral activity of the mineralocorticoid receptor NR3C2 against Herpes simplex virus Type 1 (HSV-1) infection
Source: Sci Rep. 2018 Oct 26;8:15876. doi: 10.1038/s41598-018-34241-w (PMC6203759; doi:10.1038/s41598-018-34241-w)
Supplement: Supplementary file 1 — Supplementary Information [file 41598_2018_34241_MOESM1_ESM.pdf]

Antiviral activity of the mineralocorticoid receptor NR3C2 against Herpes simplex virus Type 1 (HSV-1) infection

Jürgen G. Haas[1], Julia Weber[1], Orland Gonzalez[2], Ralf Zimmer[2], and Samantha J. Griffiths[1*]

[1]Division of Infection and Pathway Medicine, University of Edinburgh, Edinburgh EH16 4SB, U.K.

[2]Institute for Informatics, Ludwig-Maximilians Universität München, 80333 München, Germany.

[*]To whom correspondence should be addressed: samantha.griffiths@ed.ac.uk

Supplemental Figures

Figure S1

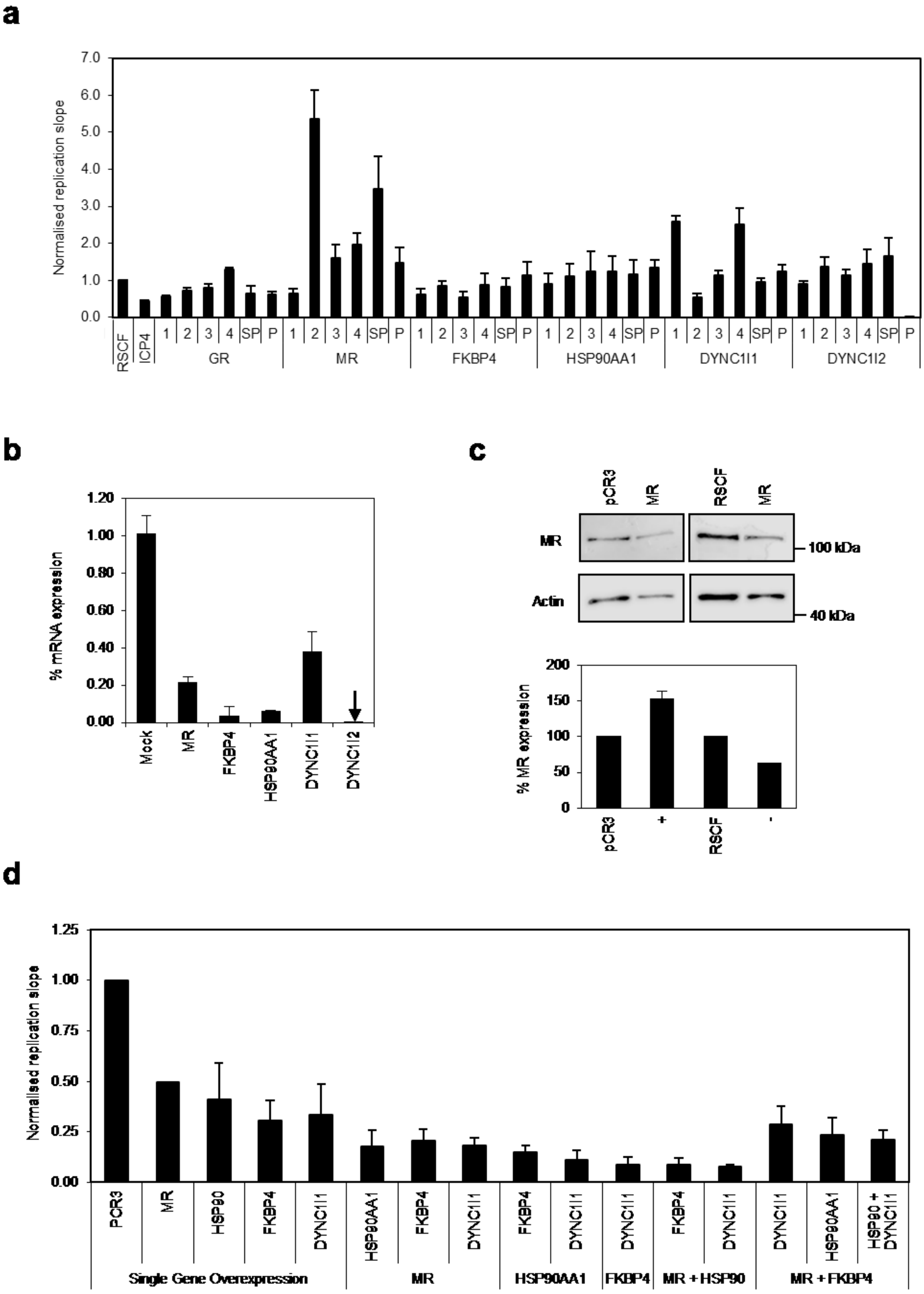

Figure S1. Validation of the MC signalling pathway as anti-viral to HSV-1. (a) Validation of siRNA screen phenotypes by siRNA deconvolution. Constituent SMARTpool siRNAs were transfected individually (1-4), and as a reconstituted SMARTpool (SP) into 293T cells, and resultant replication slopes compared to the negative control (RSCF), positive control (ICP4) and the primary screen data (P). The replication phenotype was considered validated if 2 or more of the individual siRNAs gave the same, or better, replication slope. Error bars represent the standard error of the mean of three independent experiments done in triplicates. (b) RT-qPCR confirmation of gene depletion. Gene depletion by siRNAs was confirmed in HeLa cells by RT-qPCR, normalised to the housekeeping gene hypoxanthine phosphoribosyltransferase 1 (HPRT), and relative % mRNA expression in comparison to mock-transfected cells calculated. Gene depletion of at least 60% was observed for all siRNAs tested. Error bars represent standard deviation of technical replicates from a representative experiment. (c) MR protein expression following gene overexpression or depletion. MR was overexpressed (plasmid transfection) or depleted (siRNA transfection) in HeLa cells before MR protein expression was detected by western blot and quantified in ImageStudio following Licor imaging. Band intensity signals ranged from 5,744-79,438 (actin) and 5,521-58,111 (MR). MR expression was normalised to actin. Presented blots and MR expression levels are representative of three experiments carried out in duplicates. Images have been cropped from larger gels for clarity and conciseness, presented as **Fig. S6**, but were run on the same blot and derived from the same experiment. (d) Co-expression of MC signalling pathway members synergistically inhibits HSV-1 replication. 293T cells transiently over-expressing constituent members of the MC signalling pathway alone or in combination were infected with HSV-1-eGFP at MOI 0.5 and replication monitored by GFP fluorescence. Slopes over the linear phase or replication were calculated and normalised to controls (pCR3-transfected cells). Error bars represent the standard error of the mean of at least three independent experiments, carried out in triplicates.

Figure S2

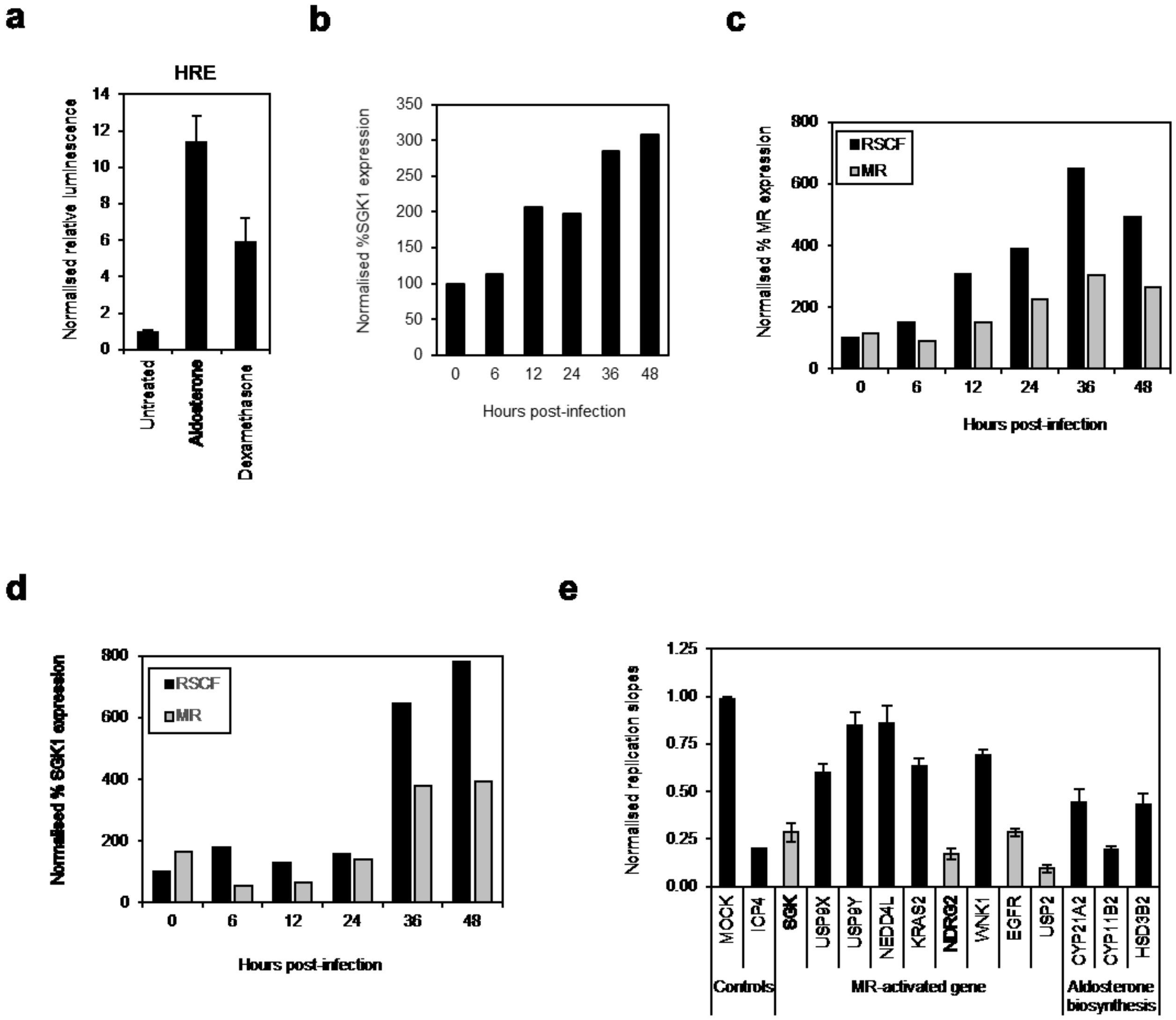
a
HRE
Normalised relative luminescence
14
12
10
8
6
4
2
0
Untreated
Aldosterone
Dexamethasone
b
Normalised %SGK1 expression
350
300
250
200
150
100
50
0
0
6
12
24
36
48
Hours post-infection
c
RSCF
MR
Normalised % MR expression
800
600
400
200
0
0
6
12
24
36
48
Hours post-infection
d
RSCF
MR
Normalised % SGK1 expression
800
600
400
200
0
0
6
12
24
36
48
Hours post-infection
e
Normalised replication slopes
1.25
1.00
0.75
0.50
0.25
0.00
MOCK
ICP4
SGK
USP9X
USP9Y
NEDD4L
KRAS2
NDRG2
WNK1
EGFR
USP2
CYP21A2
CYP11B2
HSD3B2
Controls
MR-activated gene
Aldosterone biosynthesis

Figure S2. Expression of MR and MR-regulated genes in response to HSV-1 infection. (a) Activation of the HRE by mineralocorticoid and glucocorticoid ligands. HeLa cells were transfected with HRE-luc and stimulated with the synthetic glucocorticoid dexamethasone (1 μM) or the mineralocorticoid aldosterone (5 μM) for 24 hr before activation of the HRE was determined by quantification of luciferase activity. Error bars represent the standard error of the mean of at least three experiments carried out in triplicates. (b) SGK1 expression increases at the protein level in response to HSV-1 infection. HeLa cells were infected with HSV-1 (MOI 1), and SGK1 detected by western blot in samples harvested at 0, 6, 12, 24, 36, and 48 h post-infection was normalised to actin at each time-point and expressed as a % of expression in uninfected cells (0 hr post-infection). Expression levels presented are representative of three experiments carried out in duplicates. (c) MR expression in response to HSV-1 infection when MR is depleted. HeLa cells were reverse-transfected with RSCF or MR-specific siRNA (50 nM) and, after 48 hr, were infected with HSV-1 (MOI 1). MR was detected by western blot at 0, 6, 12, 24, 36, and 48 h post-infection, was normalised to actin at each time-point and expressed as % expression in uninfected cells (0 hr post-infection). Expression levels are representative of two experiments carried out in duplicates. (d) SGK1 expression in response to HSV-1 infection when MR is depleted. HeLa cells were reverse-transfected with RSCF or MR-specific siRNA (50 nM) and, after 48 hr, cells were infected with HSV-1 (MOI 1). SGK1 was detected by western blot in samples harvested at 0, 6, 12, 24, 36, and 48 h post-infection, was normalised to actin at each time-point and expressed as % expression in uninfected cells (0 hr post-infection). Expression levels are representative of two experiments carried out in duplicates. (e) Transcriptional targets of the MR are largely pro-viral. HeLa cells were reverse-transfected with siRNA SMARTpools against transcriptional targets of the MR and infected with HSV-1-eGFP (MOI 0.5) after 48 hr. Replication was monitored as GFP fluorescence over multiple rounds of replication and slopes over the linear phase were calculated and normalised

to controls. Error bars represent the standard deviation of three experiments carried out in duplicates.

Figure S3

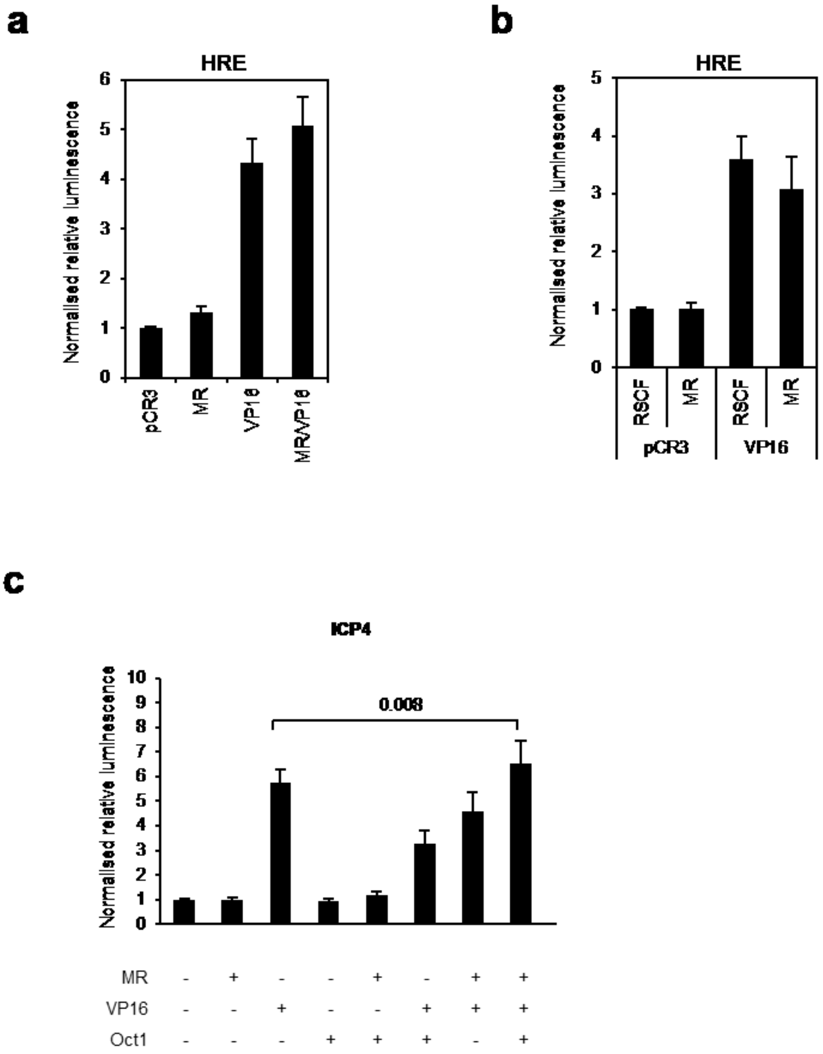

Figure S3. Modulation of HRE activation by the MR. (a) Co-expression of the MR with VP16 enhances activation of the HRE. The MR and VP16 were overexpressed in HeLa cells alone or together, with a HRE-luc reporter. After 24 hr, luciferase activity was measured and normalised to control transfected cells (pCR3). Error bars represent the standard error of the mean of four independent experiments carried out in triplicates. (b) Depletion of the MR reduces VP16-mediated activation of the HRE. HeLa cells were reverse-transfected with 20 nM control (RSCF) siRNA or a siRNA targeting the MR. The next day, cells were transfected with a HRE-luciferase reporter, and luciferase activity measured and normalised to control

transfected cells after 24 hr. Error bars represent the standard error of the mean of four independent experiments carried out in triplicates. (c) The viral IE promoter ICP4 is unaffected by co-expression of MR, VP16 and Oct1. The MR, VP16 and Oct1 were transfected into HeLa cells alone or in combinations with the ICP4-luciferase reporter. After 24 hr, luciferase activity was measured and normalised to control transfected cells (pCR3). Error bars represent the standard error of the mean of at least three independent experiments carried out in triplicates. Raw data is provided in **Table S8**.

Figure S4

a

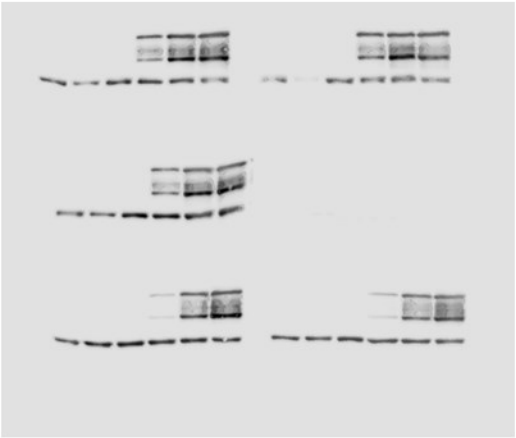

b

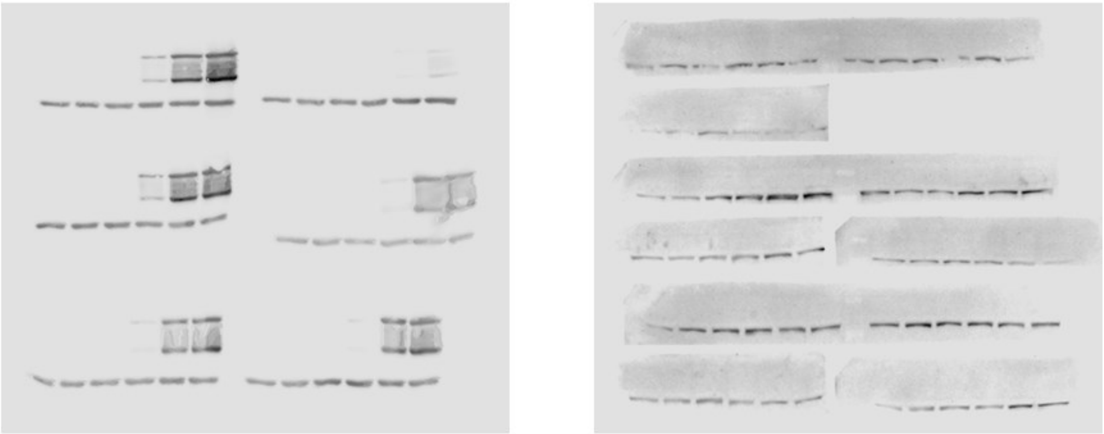

c

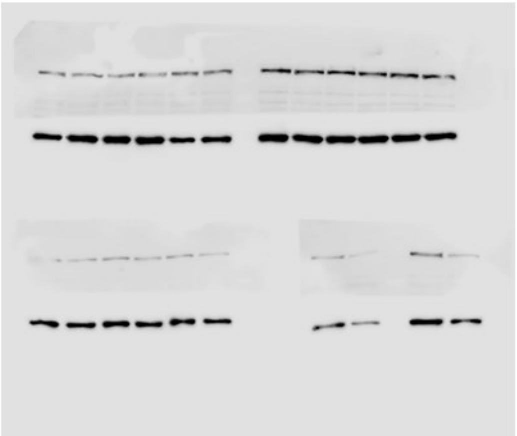

Figure S4. Full-length western blot gel images. (a) Full-size gel used for cropped images presented in Fig. 5c, Fig. 7b and Fig. S5b. (b) Full-size gel used for cropped images presented in Fig. 5d. (c) Full-size gel used for cropped image presented in Fig. S2c.

Figure S5

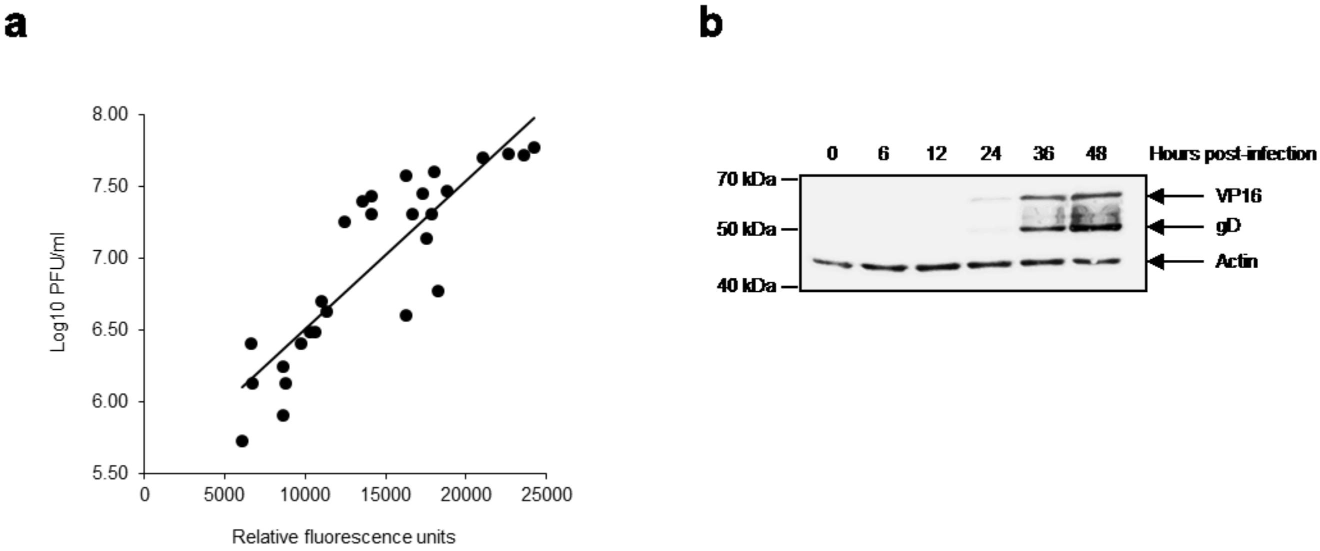

Figure S5. Replication of HSV-1-eGFP strain C12 correlates with eGFP expression. (a) Correlation between plaque-forming units and GFP fluorescence. A range of genes were depleted in HeLa cells by siRNA knockdown, before infecting with HSV-1 C12 at MOI0.5. GFP fluorescence was measured over time, and supernatant harvested for quantification of viral titre by plaque assay. Plaque forming units (PFU/ml) were correlated with GFP fluorescence. (b) Confirmation of viral gene expression following infection with HSV-1-eGFP strain C12. HeLa cells were infected with HSV-1-eGFP strain C12 at MOI1, and cells harvested at 6, 12, 24, or 48 hr post-infection. Lysates were run on Western blot and stained for immediate-early (VP16) and early (gD) viral genes, with actin staining for semi-quantitation. Image has been cropped from a larger gel, presented as **Fig. S6**, for clarity and conciseness.

Supplemental Tables

Functional Family	Gene Symbol	Mean Cell Viability	Standard Deviation
General Transcription Factors:	GTF2A2	1.00	0.03
	GTF2B	1.13	0.08
	GTF2E1	1.11	0.05
	GTF2E2	1.13	0.03
	GTF2F1	1.09	0.01
	GTF2F2	1.00	0.20
	GTF2H2	1.01	0.08
	GTF2H4	0.96	0.07
	GTF3A	1.09	0.12
	GTF3C1	1.11	0.13
	GTF3C2	0.97	0.00
	GTF3C3	0.87	0.07
	GTF3C4	0.94	0.01
	GTF3C5	1.05	0.02
Chromobox proteins:	CBX1	*0.68	0.36
	CBX3	1.09	0.03
	CBX4	0.94	0.04
	CBX5	1.04	0.05
	CBX6	0.98	0.02
	CBX8	1.12	0.04
Homeobox proteins:	HOXA1	0.99	0.02
	HOXA10	1.10	0.08
	HOXA11	1.06	0.15
	HOXA13	0.90	0.06
	HOXA2	0.93	0.10
	HOXA4	0.95	0.17
	HOXA5	0.95	0.10
	HOXA7	1.00	0.12
	HOXA9	1.11	0.03
	HOXB1	1.07	0.04
	HOXB13	1.02	0.11
	HOXB2	1.12	0.08
	HOXB3	1.22	0.03
	HOXB5	0.92	0.29
	HOXB6	0.96	0.34
	HOXB7	1.05	0.22
	HOXC10	0.97	0.25
	HOXC11	1.06	0.29
	HOXC13	1.01	0.25
	HOXC4	1.18	0.10
	HOXC5	0.96	0.10
	HOXC6	1.12	0.15
	HOXC9	1.25	0.06
	HOXD10	0.90	0.14
	HOXD11	1.02	0.12
	HOXD12	0.82	0.21
	HOXD13	1.00	0.14
	HOXD3	0.91	0.10
	HOXD4	1.11	0.11
	HOXD8	1.04	0.12

	HOXD9	1.02	0.12
Steroid hormone receptors:	NR0B1	1.10	0.04
	NR0B2	1.08	0.01
	NR1D1	0.91	0.15
	NR1D2	0.82	0.15
	NR1H2	0.83	0.15
	NR1H3	1.01	0.03
	NR1H4	1.04	0.06
	NR1I2	1.05	0.01
	NR1I3	1.08	0.03
	NR2C1	1.13	0.03
	NR2C2	1.12	0.01
	NR2E1	1.29	0.06
	NR2E3	0.97	0.03
	NR2F1	1.07	0.06
	NR2F2	1.00	0.08
	NR2F6	1.02	0.07
	NR3C1	0.99	0.04
	NR3C2	1.15	0.07
	NR4A1	1.13	0.06
	NR4A2	0.93	0.05
	NR4A3	1.18	0.05
	NR5A1	1.13	0.06
	NR5A2	0.96	0.02
	NR6A1	1.04	0.03
Mineralocorticoid signalling pathway members:	FKBP4	1.00	0.04
	FKBP5	1.00	0.07
	HSP90AA1	1.22	0.09
	DYNC1I1	1.17	0.03
	DYNC1I2	0.88	0.09

Table S1. Effect of gene depletion on HeLa cell viability. *, cell viability falls below the 70% cut-off (0.7).

Gene Symbol	siRNA 1	siRNA 2	siRNA 3	siRNA 4
General Transcription Factors:				
GTF2A2	GAAACAGUCUUCAGGAGAG	GCGAUAAUGUGUGGACUUU	GAGGUGACAGAACUUAUUA	CUAAAUACGUACAGAUUCU
GTF2B	GAAAUUGGUCGGUGUUUUA	ACAAUCAGACAGUCCUAUA	GCUAGAAACCAGUGUGGAU	ACAUAUAGCCCGUAAAGCU
GTF2E1	GAACUUGGCCUAUGAAAUA	UAACAUGGAUGACCAAGAA	CAACCGGGCUUCCUUCAAA	GGAGACAAGUUUAUCAAAU
GTF2E2	GACCAGAUACUAUUUGUAA	GCAUGACCAGCGAGGAUUA	GCUUUAAGACUCAUAACGA	AGUACAACGUGAGAGAUAA
GTF2F1	GCAAGAUGAUCAACGACAA	GAGAACACGUCCUACUACA	GAAGAAGUACGGCAUCGUC	GAAUACGUCGUUCGAGUUC
GTF2F2	GAAAGAAGACGGAAAGCGA	UAGUCAAGGUUCCUAAAUA	CAUCAGAUAAGCUGUCAUU	AGUCAGUGCUCCUAGAGAA
GTF2H2	AGACUGACGUGUACUUUAA	GGAAUGAUGCGCCACCUUU	ACAUACAAGUCGAGAAGUA	GCGAUCCAUCUAAUAUUUA
GTF2H4	GCUCAGCUCUGGUACUUUA	GAACCGAGUACACCUACAA	CUGAGGGUGUCCUGUAUAA	GCUGUAGCUCUGUGGGUAA
GTF3A	GAAACAAGGGCAAGGCUUA	CCAUAAAGAGGAAAUACUA	GCAAUGAAACAAAGUCUCA	GACCAUUUGUUUGUGACUA
GTF3C1	GAAGGUCUCUUGCGAUUGU	GACCGAAACCGGAGAGUGA	GAACGGAGAACGAUAAAGC	GUACAAGAGGCGUUACAUU
GTF3C2	UCUGUGGGAUUCAUUAUAU	CGACUGGCUUGGGACAAUA	CUUCAAUGGUGCAAAGCUA	GAGCCCACUUCAAUGCUAU
GTF3C3	GAUGUUACUUCUGCUAUUA	GAAUGUUACCUGCACUAUA	GUCCGGAACUCAUCGACUA	GCCCAAGUUUGUUUGAUAU
GTF3C4	GAAGUGUAGUUGCAGCUUA	CGACUGGAUUAAGAGGUUA	GAAUGGAACAGUCUAUACU	GAUUGGACAUAUCUCAAAG
GTF3C5	GCAGAUGUUCUACCAGUUA	GCAAGCAUACGUCAAUGUA	CGAAUCCGUUGUGGAAUGA	ACUCCGAGGUCACAUUUGA
Chromobox proteins:				
CBX1	GCGCAAAGCUGAUUCUGAU	GGAAGGGAUUCUCAGAUGA	CAGAAGAGAACCUGGAUUG	GCCCACAGGUUGUCAUAUC
CBX3	UCAGAAAGCUGGCAAAGAA	AGAAUUGAUUGAAGCGUUU	UGAAGAAUUUGUCGUGGAA	AGUACUAGAUCGACGUGUA
CBX4	GCAAGAAGCACCACCAGUA	GCUGGUCGCCCAAAUAUAA	UGGAGUAUCUGGUGAAAUG	AGACCUGGAUGAACCCAUA
CBX5	GGAUUGCCCUGAGCUAAUU	CAAGUGGAAUAUCUACUGA	AGAGAGAGCAGAGCAAUGA	UAGACAGGCGCGUGGUUAA
CBX6	GAAAGGGACGCAUCGAGUA	ACACAGAUCCGCCACAUGA	GGCCGAAUCCAUCAUCAAA	GAUGUGCAUUUCUCUGUCA
CBX8	GGAAAGGACGCAUGGAAUA	GGACGUGACCUCAAACUUU	UAACACGGACCAAGGCUUU	GCUCGCAGCCUUUGAGGAA
Homeobox proteins:				
HOXA1	GAUUACAACUUUCCAGUCG	GAACUUCAGUGCGCCUUAC	GACGACCGCUUCCUAGUGG	ACGAGAAGGCCGAGGAAUC
HOXA10	CCAACUGGCUCACGGCAAA	GCAAAGCCUCGCCGGAGAA	UCACAGCCAACUUUAAUUU	GCAAAGAGUGGUCGGAAGA
HOXA11	UAACAGAGACCGUUUACAG	GCGUCUACAUUAACAAAGA	CAGCAAAUCCACUCCUCUA	GCUCCUCUAACAUGUAUUU
HOXA13	GGGAAUACGCCACGAAUAA	GCGGACAAGUACAUGGAUA	AAGAAGCGCGUGCCUUAUA	GACAAACGGAGGCGGAUAU
HOXA2	GGAGCUGGCCUAAACAAUG	GGAUUUGACUGAGAGACAA	GAGAACUGCUUACACCAAC	CCGUAGAUAUUUCAGCUGA
HOXA4	GCAAGGAGCCCGUGGUGUA	AAGAUGCGAUCCUCCAAUU	CUACAUCGAGCCCAAGUUC	GCCCACACGCUCUGUUUGU
HOXA5	CAUAAGUCAUGACAACAUA	GCAGAAGGAGGAUUGAAAU	CGGACUACCAGUUGCAUAA	GCAGGUACGGCUACGGCUA
HOXA7	GAAAGAGCAUAAGGACGAA	GUUCCGGGCUUAUACAAUG	CAAAGGCGCCUGCGACAAG	CGUAUUAUGUGAACGCGCU
HOXA9	GCGCCGACGCCGCGGAUGA	GCGGAUGAGCUGAGCGUUG	GCAACUACUACGUGGACUC	AGUCCAAGGCGACGGUGUU
HOXB1	GCUACGGGCCUUCUCAGUA	GGAGAUGCCUCAGACCAGU	UGAAACACAGGUCAAGAUU	UCGACUGGAUGAAGGUUAA
HOXB13	GAACAGCGCUACCCCUUAA	CCACUGAGUUUGCCUUCUA	GAACCCACCAGGUCCCUUU	GUAUGCGGCUAACAAGUUC
HOXB2	CGAGUUCCCUUGGAUGAAA	GCCUUUAGCCGUUCGCUUA	GAGGGAGAUUGGGUUUAUA	CAAGGAGUCGACAUUAAUU
HOXB3	CCAAGAAGCGCCCAAAUUA	AGGCAAACGUCCAAGCUGA	GGUAAAGCCCACCAGAAUG	GCCACUAGCAACAGCAGUA
HOXB5	GCUCUUACGGCUACAAUUA	UGAGGAAGCUUCACAUCAG	GGGCAGACUCCGCAGAUAU	GCGUAUACCCGCUACCAGA

HOXB6	CGGACCCGCUGAGACAUUA	CGGAUGAAUUCGUGCAACA	UGACGGAGAGGCAGAUCAA	GCGCAGGACAAGAGCGUGU
HOXB7	GAACAAACUUCUUGUGCGU	GCGCCAAGGAGCAGAGGGA	ACUUGGCGGCCGAGAGUAA	GAGAGUAACUUCCGGAUCU
HOXC10	GAGAUUAGCAAGACCAUUA	GAGAAUGUCUGCUGCAUGU	GCAAAGUGAGUUUCCCUGA	CGGAUAACGAAGCGAAAGA
HOXC11	CAACGUGUAUAUCAACAAA	GCGCUGCCCUUAUUCGAAA	GCAAGUGGCACCAUCGGAA	CGGGAAAUCCUCUGCUGUA
HOXC13	GCAAGAAACGCGUGCCCUA	GUGACGACCUGUCCUCUAG	GCAAAUCGAAAGCGCCUCA	AGGAAUACGCGGCUAGCAA
HOXC4	CUACAUCGAUCCGAAAUUU	GCAAGCAACCCAUAGUCUA	GGACAUUACCAGGUUAUAA	CGUAUUUGAUGGACUCUAA
HOXC5	GGAUUGGACUUAAGCAUCA	CCAAUAUCCCUGCCUAUAA	CUACGUAGCCAAUUCAUUC	UGACCAAACUGCACAUGAG
HOXC6	CUAUGGAUCUAAUUCCUUU	AAGCCAGUAUCCAGAUUUA	GACAUGCUCUCAAACUGCA	CCUAUGAUCCAGUGAGGCA
HOXC9	GCUCAUCUCUCACGACAAU	GUGCCGGACUGUAGCGAUU	GACACGCGCUACAUGCGGA	GGGCCCAUCAGUAACUAUU
HOXD10	CGAAUAGAGCAACCUGUUA	GAGAUCAGUAAGAGCGUUA	UUAUAUACCUCAAGUAGAC	AAACCCAAGAGUACAAUAA
HOXD11	GAGAAGAGCAGCAGCGCAG	GAGCGCAGCCAGCAUGUAC	CGUCUGACUUCGCUAGCAA	CAACCGUCGUCCUGCCAGA
HOXD12	CCGAAGAGCAGGCUAAGUU	UCUCAAAGCGGCCAAGUAU	CUACAGAGCGGGCUAUGUG	GGAGUUGGAGAACGAAUUC
HOXD13	GAACCUAUCUGAGAGACAA	ACACCAAACUGCAGCUUAA	GAACGAGUAUGCCAUUAAC	GGACAUGUGCGUCUACCGA
HOXD3	CGACAGAACUCCAAGCAGA	GCGCGCAGCUGGUGGAAUU	GGAGAGAGCUGCGAGGACA	GCAACUUCGUCGAGUCCAU
HOXD4	UGAAAUCGCUCACACCCUG	ACACGGACCUGACGACCUU	GGUCAUGAGUUCGUAUAUG	CAACUACACCGGUGGGGAA
HOXD8	CCGAAGGCCUGACAAAUUA	AAUCAGAGCUCGUCUCCUU	UUACGGAUACGAUAACUUA	AGGCCGAGCUGGUACAAUA
HOXD9	GAAUUCCUCUUCAACAUGU	CCAAAUACCAGACGCUUGA	GAGUUCGCCUCGUGUAGUU	GCUACGAGGUGGCCAGGAU

Steroid hormone receptors:

NR0B1	CAGCAUGGAUGAUAUGAUG	CUGCUGAGAUUCAUCAAUG	ACAGAUUCAUCGAACUUAA	GAACGUGGCGCUCCUGUAC
NR0B2	GAAUAUGCCUGCCUGAAAG	GGAAUAUGCCUGCCUGAAA	CGUAGCCGCUGCCUAUGUA	GCCAUUCUCUACGCACUUC
NR1D1	GCAUGGACGCAGUGGGCGA	GGGCAUGUCUCGAGACGCU	CGGCAGGGCAACUCAAAGA	GGGCGAACGGUGCAGGAGA
NR1D2	GAAGAAUGAUCGAAUAGAU	GAACAUGGAGCAAUAUAAU	GAGGAGCUCUUGGCCUUUA	UAAACAACAUGCACUCUGA
NR1H2	CUAAGCAAGUGCCUGGUUU	GCUAACAGCGGCUCAAGAA	AGGCGAGGGUGUCCAGCUA	GAAGAAGAUUCGGAAACAG
NR1H3	GAACAGAUCCGCCUGAAGA	GAGUUUGCCUUGCUCAUUG	UGACUUUGCUAAACAGCUA	CAAGGGAGCGCACUACAUC
NR1H4	CAAGUGACCUCGACAACAA	GAAAGAAUUCGAAAUAGUG	CAACAGACUCUUCUACAUU	GAACCAUACUCGCAAUACA
NR1I2	GAUGGACGCUCAGAUGAAA	CAACCUACAUGUUCAAAGG	CAGGAGCAAUUCGCCAUUA	GCUCAUAGGUUCUUGUUCC
NR1I3	CCUCUUCGCUACACAAUUG	GAACAGUUUGUGCAGUUUA	UUAAUGCGCUGACUUGUGA	GUGGAAAUCUGUCACAUCG
NR2C1	GGAAGGAAGUGUACACCUA	GAGCACAUCUUCAAACUAC	GGAUCAAAGGAUUGUAUUA	UCUCAGCGAUUCACAUGUA
NR2C2	CUGAUGAGCUCCAACAUAA	CAACCUAAGUGAAUCUUUG	GAAGACACCUACCGAUUGG	GCGCCAAGCAACUCAUAUU
NR2E1	GAUCAUAUCUGAAAUACAG	CAAGACUGCUUUCAGAUAU	GUUAGAUGCUACUGAAUUU	CAAUGUAUCUCUAUGAAGU
NR2E3	GAGAAGCUCCUUUGUGAUA	GAAGCACUAUGGCAUCUAU	GAAGGAUCCUGAGCACGUA	GAAGCUCCUUUGUGAUAUG
NR2F1	GAAACUCUCAUCCGCGAUA	UCUCAUCCGCGAUAUGUUA	GGAACUUAACUUACACAUG	GCAAACUGCUGCUGCGACU
NR2F2	GUACCUGUCCGGAUAUAUU	CCAACCAGCCGACGAGAUU	ACUCGUACCUGUCCGGAUA	GGCCGUAUAUGGCAAUUCA
NR2F6	CGACGCCUGUGGCCUCUCA	CAGCCGGUGUCCGAACUGA	CAACCGUGACUGCCAGAUC	GUACUGCCGUCUCAAGAAG
NR3C1	GAUAAGACCAUGAGUAUUG	GGACAGAUGUACCACUAUG	GAGGACAGAUGUACCACUA	GAACUUCCCUGGUCGAACA
NR3C2	GCAAACAGAUGAUCCAAGU	CAGCUAAGAUUUAUCAGAA	GGUAUCCGGUCUUAGAAUA	GACCUAGUCUUUAAUGAAG
NR4A1	GAAGGAAGUUGUCCGAACA	CGGCUACACAGGAGAGUUU	UCGAGGACUUCCAGGUGUA	GGACAGAGCAGCUGCCCAA
NR4A2	CCACGUGACUUUCAACAAU	ACAUUCAGAUGCACAACUA	GGACAAGCGUCGCCGGAAU	CCACCUUGCUUGUACCAAA

NR4A3	CAAAGAAGAUCAGACAUUA	GAAGUUGUCCGUACAGAUA	CGGAAUACACCACGGAGAU	CCUCCAAUCUGCAUGAUGA
NR5A1	GAUUUGAAGUUCCUGAAUA	GGAGCGAGCUGCUGGUGUU	GGAGGUGGCCGACCAGAUG	CAACGUGCCUGAGCUCAUC
NR5A2	CCAAACAUAUGGCCACUUU	UCAGAGAACUUAAGGUUGA	GGAUCCAUCUUCCUGGUUA	CAUAAUGGGCUAUUCAUAU
NR6A1	CAACGAACCUGUCUCAUUU	GAAGAACUACACAGAUUUA	GAAGAUGGAUACGCUGUGA	CCGAGGACCUGGAACCAUU
Mineralocorticoid signalling pathway members:				
FKBP4	GAGCAGACCUUUAUGUAUU	CGAAAGAGCUAAAUAUCGA	GAAGUUGAGUUGAUGAAAG	GAAGAGAUCACCGGCGUAA
FKBP5	GGACGUGGUUGUCGAUUUG	GCUAGGACAUUUCAACAGA	CGACAUCAAUCAGCUAUAU	CAGACAAACUUGGGUUCUA
HSP90AA1	GCAGAUAUCUCUAUGAUUG	GAAGUGAUCUAUAUGAUUG	UAUAAGAGCUUGACCAAUG	GGAUCUCCCUCUAAACAUA
DYNC1I1	GGAAAUUCGUGCUAACAGA	CAAGGGAAGUAGUGUCCUA	GACAAUCGCAGUCAUCGAA	CGGGAGACGUCAAUAACUU
DYNC1I2	GUAAAGCUUUGGACAACUA	GAUGUUAUGUGGUCACCUA	GCAUUUCUGUGGAGGGUAA	GGGAUAACCGUAGCAAUAA

Table S2. siRNA sequences.

Gene symbol	Forward[a]	Reverse[a]	UPL Number[b]
HPRT	TGACCTTGATTTATTTTGCATACC	CGAGCAAGACGTTCAGTCCT	73
NR3C2	TTTTCTTCAAAAGAGCAGTGGA	GGACAATTCTTTCGTCGAATCT	SYBR
NR3C1	TCCCTGGTCGAACAGTTTTT	GCTGGATGGAGGAGAGCTTA	45
FKBP4	CGGGAGAAGAAGCTCTATGC	GGTCTCCTGAGGAAGCCTCT	SYBR
HSP90AA1	GTCCTGTGCGGTCACTTAGC	AAAGGCGAACGTCTCAACC	SYBR
DYNC1I1	CTGCAGTGGGACACAGACC	TTTGACACGCCCAGTTTATG	SYBR
DYNC1I2	GCATGGGGAGATTGGATTTA	ATGGGTCCATCTCACACGAT	15
SGK1	TCCTAGACTACATTAATGGTGGAGAGT	ATAGAAACGAGCCCGTGGTT	38
IFN-β	CTTTGCTATTTTCAGACAAGATTC	GCCAGGAGGTTCTCAACAAT	25
Mx1	GAAAGAGGCGAAGCGAGAG	CCGTGACACTGGGATTCCT	67
ICP4	ATGGGGTGGCTCCAGAAC	CTGCCGGTGATGAAGGAG	38
UL23	CAACAAAAAGCCACGGAAGT	CGTCTATATAAACCCGCAGTAGC	1
gC	GAGGGTCAGCCGTTCAAG	AACTCCACGGGGTTACGC	70

Table S3. Primer sequences for RT-qPCR. Primer:probe assays were designed in the Assay Design Center at http://www.universalprobelibrary.com and purchased from Roche.

[a]Primer sequences are 5' to 3'.

[b]UPL number, probe number from the Roche human Universal Probe Library. SYBR, qPCR carried out with SYBR green reagents.

		0	0.25	0.5	1	3	5	10
Exp 1	RSCF	174	176	193	206	286	275	298
		172	165	200	188	369	389	208
		145	160	229	204	346	802	376
	MR	100	107	90	108	138	144	133
		165	107	73	108	149	136	102
		102	163	87	113	177	181	144
Exp 2	RSCF	255	313	963	527	604	520	491
		150	469	374	373	634	550	461
		253	361	323	511	338	342	531
	MR	181	228	379	448	396	364	474
		160	332	238	301	409	349	403
		233	265	249	250	407	446	392
Exp 3	RSCF	188	243	385	457	753	548	477
		182	171	393	817	644	713	678
		237	359	326	485	568	695	597
	MR	162	389	309	316	583	384	502
		170	198	467	445	764	906	614
		173	328	360	552	830	805	636
Exp 4	RSCF	260	218	393	494	426	418	408
		353	233	297	335	475	587	380
		316	347	383	456	600	405	572
	MR	310	305	246	278	391	290	288
		293	275	275	298	384	265	414
		301	384	254	296	314	391	347
Exp 5	RSCF	346	394	-	364	352	468	
		239	253	-	286	390	469	
		274	312	-	416	543	477	
	MR	291	291	-	300	479	209	
		234	249	-	246	216	374	
		213	272	-	243	225	206	
Exp 6	RSCF	145		1219	1269		4021	
		186		619	2278		5207	
		149		1087	2643		6982	
	MR	125		720	2065		3357	
		171		1228	1271		5287	
		185		561	2150		5010	
Exp 7	RSCF	241	2616	5292	6171	1239	3598	993
		221	2523	3581	5260	457	2180	965
		363	3012	3262	8079	818	2423	915
	MR	242	2419	3660	6303	584	2571	1171
		166	789	1335	203	276	210	160
		180	167	224	185	212	474	615
	GR	214	151	176	198	213	188	173
		330	220	219	1000	224	195	151
		246	242	185	255	206	590	171

T**able S4. Raw luciferase data for Fig. 5a.** Raw luminescence data for activation of HRE-luc by HSV-1 at increasing multiplicities of infection, in control RSCF siRNA, MR-specific or GR-specific siRNA. Values represent raw relative luminescent units from seven independent experiments carried out in triplicate. Exp, experiment**.**

	pCR3	VP16	ICP4	ICP22	ICP27	ICP0
Exp 1	953	4947	3227	310	88	435
	1317	11524	4771	330	105	505
	572	4480	4481	617	749	501
Exp 2	569	1866	1784	134	211	358
	340	2575	2668	1211	274	532
	669	2436	3802	1101	1267	450
Exp 3	284	2634	2443	894	220	384
	463	2105	3009	964	292	365
	353	3393	4660	991	1267	359
Exp 4	1380	2032	626	780	104	
	1105	1939	612	1021	90	
	1260	2277	869	874	85	

Table S5. Raw luciferase data for Fig. 6a. Activation of HRE-luc reporter by HSV-1 transcription factors. Values represent raw relative luminescent units from four independent experiments carried out in triplicate. Exp, experiment.

	Exp 1			Exp 2			Exp 3			Exp 4		
	pCR3	VP16	ICP4	pCR3	VP16	ICP4	pCR3	VP16	ICP4	pCR3	VP16	ICP4
**AP1**	693	296	102	390	935	1054	353	514	1868	713	304	2581
	901	272	128	473	771	1319	276	591	1814	757	316	2614
	837	180	132	523	796	3066	469	986	1407	956	411	2197
**NF-KB**	11575	6133	22173	11630	21543	26677	11070	10928	18567	24590	9426	21675
	11514	4074	15634	10224	14179	24352	11292	10339	15833	24095	8225	29099
	13140	3060	11542	14269	17880	25848	12091	16312	16510	38397	9741	36032
**IFN-B**	204	129	319	328	405	3960	258	273	2920	449	283	2053
	78	87	239	223	231	3062	199	279	2235	287	118	2467
	39	131	444	310	294	3440	183	200	3469	286	144	2368
**ISRE**	957	762	1028	553	1236	755	315	784	779	642	461	312
	879	885	1205	411	1007	944	347	564	745	606	565	214
	696	723	1215	497	1203	658	394	592	617	604	529	175
**HRE**	953	4947	3227	569	1866	1784	284	2634	2443	1380	2032	626
	1317	11524	4771	340	2575	2668	463	2105	3009	1105	1939	612
	572	4480	4481	669	2436	3802	353	3393	4660	1260	2277	869

Table S6. Raw luciferase data for Fig. 6b. Activation of a panel of luciferase promote element reporters by the HSV-1 transcription factors VP16 and ICP4. Values represent raw relative luminescent units from four independent experiments carried out in triplicate. Exp, experiment.

	pCR3<br>pCR3	MR	VP16	Oct1	Oct1<br>MR	VP16	VP16<br>MR	VP16 + Oct1<br>MR
Exp1	5469	7534	18374	13754	13680	44880	21343	37927
	4812	3858	10260	7689	9403	37975	15599	34082
	6084	6062	16765	16801	20993	35679	19473	54627
Exp2	6645	5877	17364	6182	4093	13112	10658	10959
	6201	3672	11717	2899	3982	13663	19978	10823
	9145	7806	15113	9183	7058	24129	17570	14489
Exp3	2651	769	5889	725	2354	6993	8191	24511
	3190	860	8129	2219	3017	15060	14506	23614
	2024	675	9232	1194	3165	12621	9617	18430

Table S7. Raw luciferase data for Fig. 6c. Synergistic activation of HRE-luc by MR, VP16 and Oct1. Values represent raw relative luminescent units from three independent experiments carried out in triplicate. Exp, experiment number.

	pCR3<br>pCR3	MR	VP16	Oct1	Oct1<br>MR	VP16	VP16<br>MR	VP16 + Oct1<br>MR
Exp1	177104	176303	876966	250747	359638	1098582	920930	949946
	239365	270227	879860	208415	225185	900390	1364260	1459285
	214645	286086	1181493	282201	323592	1151878	1371966	1197649
Exp2	182679	319510	1086427	326996	377673	871172	701504	1008858
	307526	306324	649425	214876	162659	486951	812683	549475
	286017	409813	1109041	343582	416750	1136559	1003496	1365851
Exp3	44250	29084	207561	29153	32917	270245	349980	604934
	69114	26785	247782	24287	22218	122779	205906	220848
	75883	38175	240338	12218	44997	212367	338951	358058

Table S8. Raw luciferase data for Fig. S3c. Synergistic activation of ICP4-luc by MR, VP16 and Oct1. Values represent raw relative luminescent units from three independent experiments carried out in triplicate. Exp, experiment number.
